# Polycarbonate-based ultra-pH sensitive nanoparticles improve therapeutic window

**DOI:** 10.1038/s41467-020-19651-7

**Published:** 2020-11-17

**Authors:** Xu Wang, Jonathan Wilhelm, Wei Li, Suxin Li, Zhaohui Wang, Gang Huang, Jian Wang, Houliang Tang, Sina Khorsandi, Zhichen Sun, Bret Evers, Jinming Gao

**Affiliations:** 1 Department of Pharmacology, Harold C. Simmons Comprehensive Cancer Center, University of Texas Southwestern Medical Center, Dallas, TX 75390 USA; 2 Department of Radiology, University of Texas Southwestern Medical Center, Dallas, TX 75390 USA; 3 Department of Pathology and Ophthalmology, University of Texas Southwestern Medical Center, Dallas, TX 75390 USA

**Keywords:** Tumour vaccines, Biomaterials - vaccines, Nanomedicine

## Abstract

Stimuli-sensitive nanomaterials with cooperative response are capable of converting subtle and gradual biological variations into robust outputs to improve the precision of diagnostic or therapeutic outcomes. In this study, we report the design, synthesis and characterization of a series of degradable ultra-pH sensitive (dUPS) polymers that amplify small acidic pH changes to efficacious therapeutic outputs. A hydrolytically active polycarbonate backbone is used to construct the polymer with pH-dependent degradation kinetics. One dUPS polymer, PSC7A, can achieve activation of the stimulator of interferon genes and antigen delivery upon endosomal pH activation, leading to T cell-mediated antitumor immunity. While a non-degradable UPS polymer induces granulomatous inflammation that persists over months at the injection site, degradable PSC7A primes a transient acute inflammatory response followed by polymer degradation and complete tissue healing. The improved therapeutic window of the dUPS polymers opens up opportunities in pH-targeted drug and protein therapy.

## Abstract (web summary)

Stimuli-responsive nanomaterials offer the opportunity to exploit nanoscale cooperativity to improve the precision of diagnostic or therapeutic outcomes. Here, the authors report the design, synthesis and characterization of a series of degradable ultra-pH sensitive polymers that amplify small acidic pH changes to efficacious therapeutic outputs.

## Introduction

pH plays an important role in a number of biological processes including endosome/lysosome maturation^1^, antigen processing^2^, protein and lipid metabolism^3^, and tumor pathophysiology^4,5^. A variety of pH-sensitive polymers and nanoparticles (NPs) have been developed for therapeutic delivery and imaging applications^6–8^. For many biological processes, pH variations are small from normal physiological pH (7.4)^9,10^. For example, extracellular pH of tumors is reported ~6.8 and early endosomal pH is at 6.5^4^. Small molecular or non-cooperative polymeric pH-sensitive systems are not efficient to respond to these subtle pH differences, which prompted successful development of various smart materials with improved pH sensitivity and response^11–13^.

Previously, our lab synthesized a series of tunable, ultra-pH sensitive (UPS) polymers with a sharp response to a predetermined pH threshold. Poly(methyl methacrylate) (PMMA) backbone was used to construct these polymers, where the sharpened pH sensitivity and tunability arise from the ionizable tertiary amines with different hydrophobic substituents^14–16^. The UPS NPs have demonstrated precision in several biological applications, including tumor imaging and surgery^17,18^, buffering of endocytic organelles and lysosome catabolism^19,20^, and T cell therapy of cancer^21^. In cancer immunotherapy, we discovered PEG-*b*-PC7A (abbreviated as PC7A), a UPS polymer with a cyclic tertiary amine of 7-membered ring, as a potent vaccine adjuvant^21^. Tumor antigens can be stably loaded inside PC7A NPs and upon subcutaneous injection, PC7A NPs quickly drain to the lymph nodes to target antigen-presenting cells. One unique feature of PC7A is its ability to bind and activate the stimulator of interferon genes (STING), which elevates the expressions of co-stimulatory molecules (CD80/CD86) on dendritic cells and rapid release of type I interferons. Consequently, PC7A nanovaccines demonstrate robust cytotoxic T cell response, with significantly improved antitumor immunity in multiple cancer models.

In this study, we report the design, synthesis, and characterization of a degradable series of ultra-pH sensitive (dUPS) block copolymers. Backbone chemistry plays a crucial role in pH sensitive response. Prior attempts using amide bonds as backbones failed to achieve sharp pH response. Polycarbonates are widely used in biological applications due to their excellent biocompatibility and biodegradability^22–26^. Upon hydrolytic degradation, polycarbonates produce small molecular alcohols and carbon dioxides, which are non-toxic and can be rapidly secreted^27^. Here we show polycarbonate-based dUPS polymers retain the ultra-pH sensitive response as the PMMA-based non-degradable polymers. Moreover, a degradable vaccine adjuvant, PSC7A polymer, is able to bind STING with high affinity. PSC7A nanovaccines achieve efficacious tumor growth inhibition with prolonged animal survival reliant on STING. The degradable PSC7A also displays significantly improved long-term safety as indicated by the lack of granuloma formation compared to the non-degradable PC7A polymer. Overall, dUPS polymers are able to increase the therapeutic window of T-cell vaccine immunotherapy and avoid the undesirable host response to dose escalation.

## Results

### Synthesis and characterization of dUPS copolymers

**Fig. 1:**
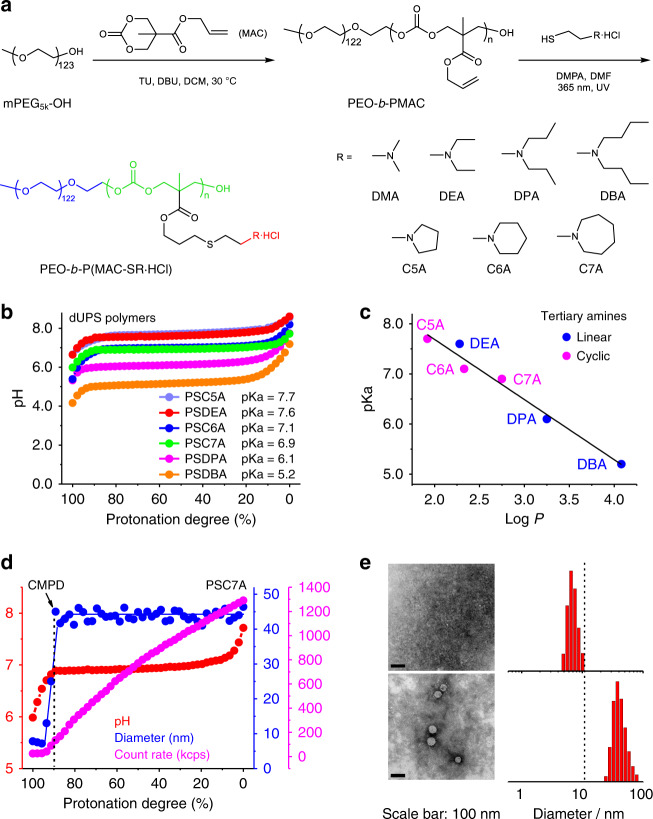
Micellization-induced ultra-pH sensitivity of degradable ultra-pH sensitive (dUPS) polymers. **a** Synthesis of PEO-*b*-P(MAC-SR·HCl) (abbreviated as PSR) by ring-opening polymerization and thiol-ene reactions. The final copolymers consist of a hydrophilic PEO segment (blue), a degradable polycarbonate backbone (green), and ionizable tertiary amines lending pH sensitivity (red). **b** dUPS polymers display ultra-pH sensitivity and strong buffer capacity at their relative pK_a_. PSDMA titration is presented separately in Supplementary Fig. 10. **c** pK_a_ values are inversely correlated with the log *P* of the repeating unit of the P(MAC-SR) segment (neutral/deprotonated state). **d** Number-weighted hydrodynamic diameters and light scattering count rates as a function of protonation degree during the PSC7A titration. CMPD: critical micellization protonation degree. **e** TEM images and corresponding number-weighted hydrodynamic diameter distributions by dynamic light scattering (DLS) analysis of PSC7A in 150 mM NaCl solution at 95% and 85% protonation degrees, above and below CMPD. Scale bar: 100 nm. TEM measurements of PSC7A polymer solutions at 85% and 95% protonation degrees were repeated three times independently with similar results, and one representative image from each group was shown.

Figure 1a illustrates the synthesis of polycarbonate-based dUPS copolymers using the ring-opening polymerization (ROP) method^28–30^. First, methoxy-terminated polyethylene glycol (mPEG_5k_-OH, *M*_n_ = 5.4 × 10^3^ g/mol as measured by ^1^H NMR) was used as a macroinitiator to react with 5-methyl-5-allyloxycarbonyl-1,3-dioxan-2-one (MAC) as the cyclic monomer in dichloromethane (DCM). 1-(3,5-bis-trifluoromethyl-phenyl)-3-cyclohexylthiourea (TU) and 1,8-diazabicyclo[5.4.0]undec-7-ene (DBU) were used as organic co-catalysts. The reaction mixture was heated at 30 °C for 15 h to yield an allyl group functionalized block copolymer, poly(ethylene oxide)-*b*-poly(5-methyl-5-allyloxycarbonyl-1,3-dioxan-2-one) (PEO-*b*-PMAC) with average 125–140 repeating units in the PMAC segment by ^1^H NMR measurement. To render pH sensitivity, PEO-*b*-PMAC was further reacted with a series of protonated tertiary amines (R∙HCl) through thiol-ene reactions under UV light (365 nm, Fig. 1a). Tertiary amines with different alkyl chain lengths and cyclic structures were used, leading to a series of biodegradable, ultra-pH sensitive copolymers PEO-*b*-P(MAC-SR∙HCl) in the protonated states (Supplementary Table 1 and Supplementary Figs. 2–8). For simplicity, we use PSR to refer to the block copolymer PEO-*b*-P(MAC-SR∙HCl) and its deprotonated state below.

### Micellization-induced ultra-pH sensitivity

pH titration of the dUPS copolymers (1.0 mg/mL in aqueous solution) was carried out in the presence of 150 mM NaCl to mimic the physiological salt concentration. Data are presented as pH over the protonation degree of the tertiary amine residues on the copolymer. The protonation degree was calculated as the molar percentage of the protonated amines over the total amine concentration. The apparent p*K*_a_ value of each copolymer was determined as the pH at 50% of the protonation degree. To evaluate the sharpness in pH transition, we also measured the ∆pH_10–__90%_, the pH range between 10% and 90% protonation degrees, for each copolymer (Supplementary Fig. 9).

Results show that except for PSDMA (abbreviated for PEO_123_-*b*-P(MAC-SDMA)_135_) with the dimethyl amine side chains, all the other copolymers displayed ultra-pH sensitive behaviors (Fig. 1b). The ultra-pH response is represented by the remarkable pH plateau across a broad range of protonation degrees, in particular from 10% to 90%, demonstrating the strong pH buffer effect. The ∆pH_10–__90%_ values were at or below 0.5 for these copolymers. For PSDMA, a higher ∆pH_10–__90%_ value (1.2) was observed which did not exhibit ultra-pH sensitive characteristics. Commonly used polybases (e.g., poly(ethyleneimine), chitosan, polyhistidine, polylysine) display broad pH responses with ∆pH_10–__90%_ >1 (Supplementary Fig. 9)^31^.

The apparent p*K*_a_ values of the dUPS copolymers show an inverse correlation with the hydrophobicity of the tertiary amines (Fig. 1c). We used the octanol-water partition coefficients (log *P*) to quantify the hydrophobicity of the repeating unit of the P(MAC-SR) segment in its neutral/deprotonated state. Data show a coalesced correlation of the apparent p*K*_a_’s *vs*. log *P* irrespective of cyclic or linear tertiary amine structures. Similar to the non-degradable UPS polymers^32^, the dUPS polymers synthesized from monomers with higher hydrophobicity exhibited a lower p*K*_a_ value. The p*K*_a_ values of these copolymers encompassed a broad range of physiological pH from 5.2 to 7.7.

Next, we investigated the impact of hydrophobic micellization on pH sensitivity for two copolymers, PSC7A (abbreviated for PEO_123_-*b*-P(MAC-SC7A)_135_) and PSDMA. PSC7A has a broad pH plateau in the majority of the protonation degrees (Fig. 1c), whereas PSDMA only displays pH plateau when the protonation degree is <50% (Supplementary Fig. 10). For PSC7A, dynamic light scattering (DLS) results (Fig. 1d) show that the polymer chains existed as unimers (single chains) with average hydrodynamic diameter below 10 nm when the protonation degree was above 90%. Micelles began to form when the protonation degree decreased to 90% and below as indicated by the increase in scattering count rates. We defined critical micellization protonation degree (CMPD) as the protonation degree below which the polymer chains begin to self-assemble. For PSC7A, the CMPD value is at 90%. Transmission electron microscopy (TEM) images and number-weighted hydrodynamic diameter distributions of PSC7A at the protonation degrees of 95% and 85% (Fig. 1e) further corroborate micelle formation across CMPD. Micelle diameters were ~45 nm with protonation degree below 90%.

Micellization-induced ultra-pH sensitivity is more illustrative with PSDMA. The polymer displays a two-segmented pH response with a broad response above 50% protonation degree and a narrow one below 50% (Supplementary Fig. 10). The CMPD value was measured to be 50% by DLS and TEM analysis. Above 50% protonation degree, PSDMA stayed as unimers and showed a broad pH response. Below 50% protonation, a dramatically sharpened pH response was observed. These results demonstrate micelle phase transitions are responsible for the cooperative pH response of the dUPS copolymers, similar to the non-degradable UPS polymers^31^.

We performed ^1^H NMR measurements in deuterated water (D_2_O) to validate the protonation degrees defined in the titration data (Supplementary Fig. 11). Solutions of PSC7A copolymer in D_2_O at protonation degrees of 0%, 20%, 40%, 60%, 80%, and 100% were prepared by adding specified volumes of NaOD according to the pH titration data. The proton signal of PEO segment remained constant and was used as internal reference. Peaks **a** (2H, -SCH_2_C*H*_2_N-) and **b** (4H, -N(C*H*_2_CH_2_CH_2_)_2_), which were adjacent to the nitrogen atom, overlapped with each other. Their chemical shifts did not change along the pH titration process, but the integrations decreased linearly with the decreasing of the protonation degree. The decrease in the peak area reflected the loss of proton signals in the unimers due to the formation of micelles where increased transverse (T_2_) relaxation abolished the proton NMR signals in the micelle state. The NMR data agree with the divergent protonation of tertiary amine residues in the unimer versus micelle states as displayed in the previous UPS system^31^.

### STING activation and T-cell therapy of cancer

Stimulator of interferon genes (STING) is an endoplasmic reticulum (ER)-bound homodimeric protein that plays a critical role in innate immunity^33,34^. STING activation leads to upregulation of type I interferons (IFNs), which enhance the CD8^+^ T-cell response against cancer^35–37^. Previously, we reported a non-degradable polymer NP, PC7A NP, that allows efficient encapsulation of tumor antigens and cytosolic delivery to lymph node-resident dendritic cells. The polymer also binds and activates STING and turns on the co-stimulatory signals (CD80/CD86) in antigen-presenting cells for the generation of antigen-specific T cells^21^.

**Fig. 2:**
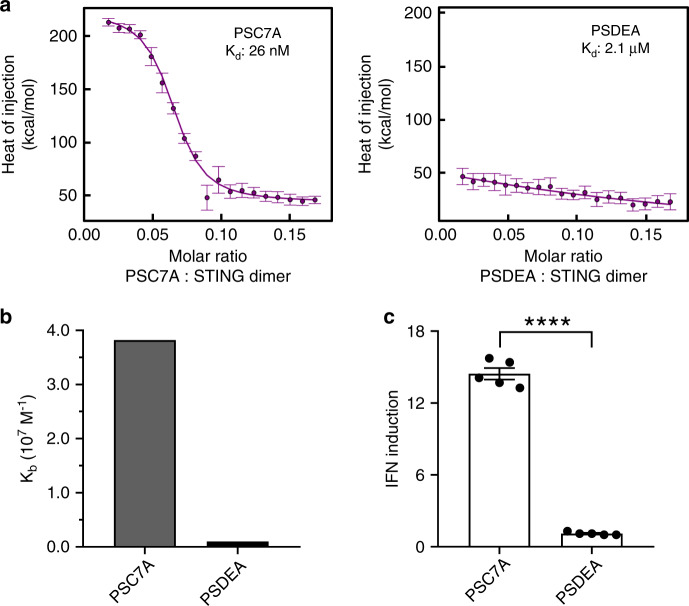
Stimulator of interferon genes (STING) binding and activation by the PSC7A polymer. **a** Isothermal titration calorimetry (ITC) analysis shows the PSC7A copolymer has a much higher binding affinity to STING than PSDEA. *K*_d_: apparent dissociation constant. The error bars are estimates of the integration error based on the noise in the baseline regions flanking each injection. **b** Summary of *K*_b_ values (binding affinity, *K*_b_ = 1/*K*_d_) of PSC7A and PSDEA to STING from ITC experiments. **c** Interferon (IFN) induction levels of THP1-ISG cells incubated with PSC7A or PSDEA (0.5 μM for 48 h) correlate with STING binding affinity. *n* = 5 experimental replicates in each group. Data are presented as means ± s.e.m. and statistical significance was calculated using Student’s two-tailed *t* test (PSC7A vs. PSDEA: ^****^P < 0.0001). Results from additional dUPS polymers are presented in Supplementary Fig. 13.

In this study, we first evaluated the binding affinity of a series of dUPS copolymers (PSC7A, PSC6A, PSC5A, and PSDEA) to the C-terminal domain of STING (139-397 AAs). While the STING protein is located in the cytosol near a pH of 7–7.4, PSC7A polymers (p*K*_a_ = 6.9) form micelles in this pH range and do not bind to STING due to PEG shielding. However, in the presence of proteins (e.g., albumin or cytosolic proteins), PSC7A was able to maintain as unimers after pH reversal from 6.5 to 7.4 (Supplementary Fig. 12). Under these conditions, significant noise from albumin perturbation and extreme sensitivity of isothermal titration calorimetry (ITC) prevented accurate measurement of binding affinity. Therefore, we chose pH 6.5 as condition to mimic the dissociated unimer state of the dUPS polymers without introducing the protein interference and quantify the binding interactions between dUPS polymers and STING. Data show that PSC7A copolymer had the highest binding affinity to STING with a dissociation constant (*K*_d_) at 26 nM (Fig. 2a and Supplementary Fig. 13). The other two copolymers PSC6A and PSC5A with cyclic tertiary amines had less binding with *K*_d_ values at 43 and 84 nM, respectively (Supplementary Fig. 13). The copolymer PSDEA with linear tertiary amines had negligible binding to STING. The *K*_d_ value of non-degradable PC7A to STING is 72 nM, higher than that by the PSC7A. We used THP1-ISG cells to evaluate STING activation after treatment by different copolymers (0.5 μM) for 48 h (Fig. 2c and Supplementary Fig. 13). THP1-ISG cells were transfected by a luciferase reporter gene under the control of an interferon regulatory factor-inducible promoter. Upon STING activation, secretion of type I IFN will activate luciferase expression for luminescent detection. Results show IFN induction was elevated with dUPS copolymers with cyclic tertiary amines over the linear analogs. In particular, PSC7A copolymer resulted in a maximum of 14-fold increase in IFN induction, which correlated with the highest binding affinity to STING by the ITC measurement.

**Fig. 3:**
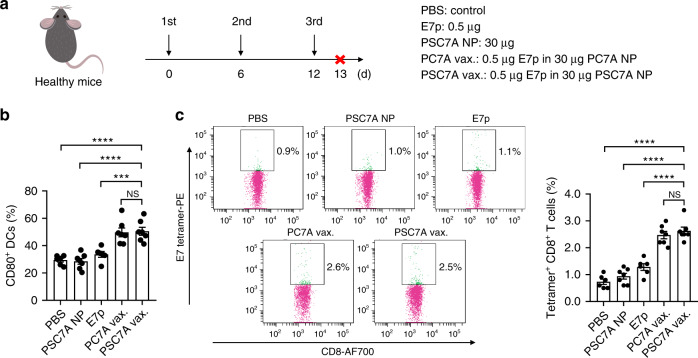
PSC7A nanovaccine increases the percentage of CD80^+^ dendritic cells and tetramer^+^ CD8^+^ T cells in vivo. **a** C57BL/6 healthy mice were treated with PBS, E7p, PSC7A NP, and PC7A vaccine (PC7A vax.) or PSC7A vaccine (PSC7A vax.) three times in 6 day intervals. One day after the last administration (on day 13), the mice were sacrificed and lymph nodes were harvested for flow cytometry analysis. **b** Summary of percentage of CD80^+^ DCs in inguinal lymph nodes 1 day after the last administration. *n* = 6 biologically independent mouse samples in each PBS and E7p group. *n* = 7 biologically independent mouse samples in each PSC7A NP, PC7A vaccine, and PSC7A vaccine group. Data are presented as means ± s.e.m. and statistical significance was calculated using Student’s two-tailed *t*-test (PSC7A vax. vs. E7p: ^***^P = 0.0008, PSC7A vax. vs. PSC7A NP: ^****^P < 0.0001, PSC7A vax. vs. PBS: ^****^P < 0.0001). **c** Representative flow dot plots of E7 tetramer staining of CD8^+^ T cells in lymph nodes and summary of percentage of E7 tetramer^+^ CD8^+^ T cells measured by flow cytometry. *n* = 6 biologically independent mouse samples in each PBS and E7p group. *n* = 7 biologically independent mouse samples in each PSC7A NP, PC7A vaccine, and PSC7A vaccine group. Data are presented as means ± s.e.m. and statistical significance was calculated using Student’s two-tailed *t* test (PSC7A vax. vs. E7p: ^****^P < 0.0001, PSC7A vax. vs. PSC7A NP: ^****^P < 0.0001, PSC7A vax. vs. PBS: ^****^P < 0.0001).

Due to its high STING activity, we chose PSC7A copolymer to generate a T-cell vaccine. The PSC7A vaccine was prepared by mixing PSC7A NPs (30 μg) with an E7 peptide antigen (E7p, GQAEPDRAHYNIVTFCCKCD, 0.5 μg). To investigate the degradability of PSC7A on antitumor efficacy, we also prepared the nanovaccine using non-degradable PC7A NPs with E7p^21^. To examine the immune response induced by the vaccine in vivo, we treated C57BL/6 mice with PBS, E7 peptide alone, PSC7A NP, PC7A vaccine or PSC7A vaccine subcutaneously at the tail base three times in 6 day intervals. One day after the last administration (on day 13), the mice were sacrificed and their inguinal lymph nodes were harvested and analyzed by flow cytometry (Fig. 3). We found a significant increase of the percentage of CD80^+^ DCs and E7 tetramer^+^ CD8^+^ T cells in both PSC7A vaccine and PC7A vaccine groups, while the difference between the PSC7A and PC7A groups was not significant, indicating a similar immune stimulation potential by both polymers.

**Fig. 4:**
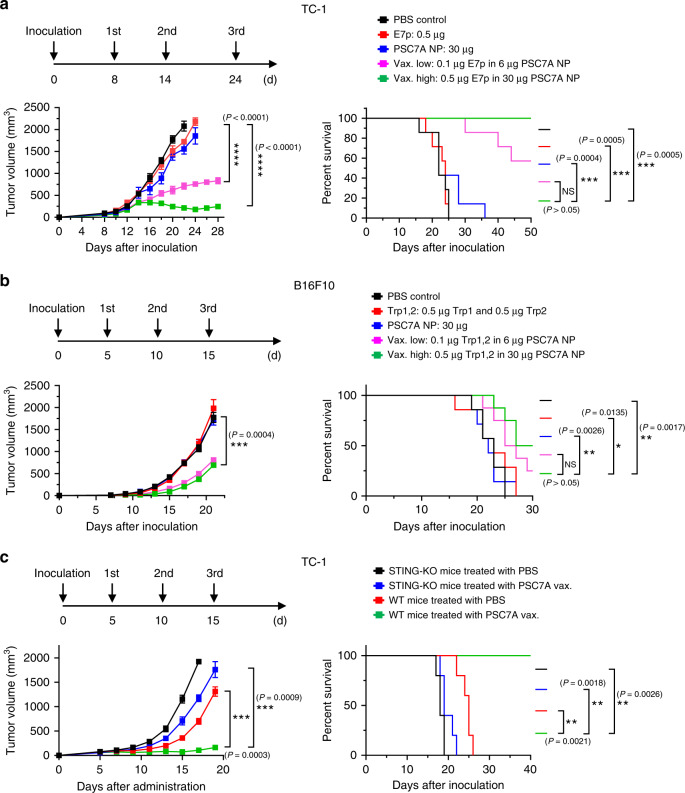
PSC7A nanovaccine inhibits tumor growth and prolongs survival in tumor-bearing mice. C57BL/6 mice inoculated with 2 × 10^5^ **a** TC-1 or **b** B16F10 melanoma cells were treated with PBS, tumor antigenic peptides only, PSC7A NP, low-dose PSC7A vaccine (Vax. low), or high-dose PSC7A vaccine (Vax. high) at specific time points, indicated above. Vaccination induced potent tumor growth inhibition and extended survival of these mice. TC-1 model: *n* = 7 biologically independent mice in each group. Tumor growth data are presented as means ± s.e.m. and statistical significance was calculated using two-way ANOVA with Dunnett’s multiple comparison testing (Vax. high vs. PBS control: ^****^P < 0.0001, Vax. low vs. PBS control: ^****^P < 0.0001, based on 22 day data after inoculation). B16F10 model: *n* = 7 biologically independent mice in each PBS, peptides only and PSC7A NP group, *n* = 8 biologically independent mice in each Vax. high and Vax. low group. Tumor growth data are presented as means ± s.e.m. and statistical significance was calculated using two-way ANOVA with Dunnett’s multiple comparison testing (Vax. high vs. PBS control: ^***^P = 0.0004, based on 21 day data after inoculation). **c** Wildtype (WT) and STING knockout (STING-KO) C57BL/6 mice inoculated with 2 × 10^5^ TC-1 cells were treated with PBS or PSC7A vaccine at specific time points, indicated above. *n* = 5 biologically independent mice in each group. Tumor growth data are presented as means ± s.e.m. and statistical significance was calculated using two-way ANOVA with Dunnett’s multiple comparison testing (WT PSC7A vax. vs. WT PBS: ^***^P = 0.0003, WT PSC7A vax. vs. KO PSC7A vax.: ^***^P = 0.0009, based on 19 day data after inoculation). For all the survival analysis, the statistical significance was calculated by the log-rank test (^****^P < 0.0001, ^***^P < 0.001, ^**^P < 0.01, ^*^P < 0.05) and the exact *P* values are indicated in the figures.

To determine the efficacy of the PSC7A vaccine in tumor treatment, we used human papilloma virus (HPV) E6/E7-transfected TC-1 and murine B16F10 melanoma tumor models. Six- to eight-week-old C57BL/6 mice were first inoculated with tumor cells (2 × 10^5^) on the right thigh. In the TC-1 model, different groups were subcutaneously injected at the tail base on day 8, 16, and 24 after tumor inoculation (indicated in Fig. 4a). PBS, E7p, and PSC7A NP only groups were used as controls. Results show that E7p and PSC7A NP only groups had marginal tumor growth inhibition response over the PBS control. Most of the animals were lost within 30 days after tumor inoculation. In contrast, the E7p-PSC7A NP groups led to dramatically improved tumor growth inhibition and prolonged survival. The low-dose PSC7A vaccine group (0.1 μg E7p in 6 μg PSC7A NP) resulted in >50% animal survival 50 days after tumor inoculation, whereas high-dose vaccine group (0.5 μg E7p in 30 μg PSC7A NP) had complete survival outcome (Fig. 4a). For the B16F10 melanoma tumor model, a combination of tumor-associated antigens (Trp1_214–237_ and Trp2_173–196_) were loaded in PSC7A NPs. Peptide-PSC7A NP groups also displayed significantly improved tumor growth inhibition and prolonged survival over the PBS control and peptide or PSC7A NP only groups (Fig. 4b). In these two models, PSC7A vaccines showed slightly improved tumor growth inhibition in the TC-1 model and similar responses in the B16F10 model compared to PC7A vaccines at the same dose, indicating the two polymers maintain similar antitumor efficacy (Supplementary Fig. 14). The PSDEA NP vaccine with E7 peptide showed drastically reduced efficacy compared to PSC7A vaccine and similar tumor growth curves as the PBS control (Supplementary Fig. 15), which provided further support for the necessity of PSC7A in antitumor therapy.

To further support the role of STING in PSC7A-induced antitumor efficacy, we performed animal studies in STING knockout (STING-KO) C57BL/6 mice. Six- to eight-week-old wildtype (WT) and STING-KO C57BL/6 mice were first inoculated with TC-1 tumor cells (2 × 10^5^) on the right thigh. Either PBS or PSC7A vaccine (0.5 μg E7p in 30 μg PSC7A NP) was subcutaneously injected at the tail base on day 5, 10, and 15 after tumor inoculation. In this study, WT mice treated with PSC7A vaccine displayed significant antitumor response compared with the other three treatment groups, with all animals surviving past 40 days. The knockout of STING abrogated antitumor efficacy of the PSC7A vaccine, leading to rapid tumor growth and shortened survival of the mice (Fig. 4c).

### Degradation studies

**Fig. 5:**
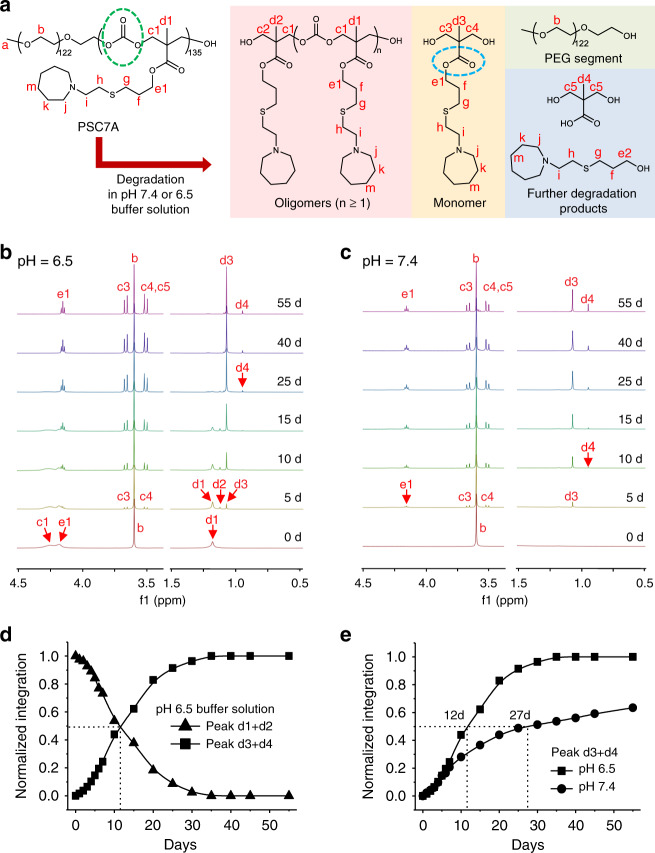
Degradation studies of PSC7A copolymer in deuterated buffer solutions at pH 6.5 and 7.4. **a** Chemical structures of PSC7A and its degraded products. ^1^H NMR spectra of the degradation of PSC7A in **b** pH 6.5 and **c** pH 7.4 deuterated buffer solutions over time. Proton signal of PEO segment was used as an internal reference. Only selected regions of the spectra are presented for clarity, see the complete spectra in Supplementary Figs. 16 and 17. **d** Normalized integration ratios of peaks (d1 + d2), and peaks (d3 + d4), relative to normalized proton signal of the PEO segment in pH 6.5 deuterated buffer solution. **e** Integration ratios of peaks (d3 + d4) relative to normalized proton signal of the PEO segment in pH 6.5 and 7.4 deuterated buffer solutions.

We investigated the PSC7A degradation properties at pH 7.4 and 6.5 that mimic the normal physiological pH and early endosomal pH environments, respectively. The apparent p*K*_a_ of PSC7A is 6.9, thereby the copolymer exists as micelles at pH 7.4 or protonated unimers at pH 6.5. The copolymer was prepared at 5.0 mg/mL in deuterated phosphate buffer solutions (50 mM) with NaCl (150 mM). NaOD or DCl solutions were added to adjust the pH to 6.5 or 7.4. Figure 5a shows the structure of the PSC7A copolymer and its degraded products. In the degradation process, the copolymer can degrade into oligomers, monomers, PEO segment, carbon dioxide, as a result of the hydrolytic cleavage of the polycarbonate backbone (green circle in Fig. 5a). Further hydrolysis of the ester groups on the side chain (blue circle in Fig. 5a) can lead to products over 55 days in both pH solutions. Proton signal of PEO segment did not change overtime, and we used its peak area as an internal reference for quantification of degradative products.

Figure 5b shows the degradation profile of PSC7A at pH 6.5 over time. Because the copolymer existed as protonated unimers in solution, all the proton peaks were visible at time zero (e.g., proton signals corresponding to c1, d1, and e1). Within the first several days, new peaks (c3, c4, d2, and d3) formed and their intensity increased over time. On day 25, it appeared a majority of the copolymers degraded into monomeric structures (yellow panel in Fig. 5a) and PEO. Additional hydrolysis from day 25 to 55 shows a small percentage of further degradation product of bis-HPA (d4). Quantification of degradation was performed by analyzing the decrease of peak intensity of d1 and d2 (from polymer and oligomer states, respectively) or the increase of d3 and d4 (monomers and bis-HPA). Data shows simultaneous crossing of the two sets of curves at 50% of the relative peak intensity on day 12 (Fig. 5d). We define *t*_1/2_ as the half-time for the conversion of 50% copolymers into monomers (*t*_1/2_ = 12 d at pH 6.5). The degradation profile at pH 7.4 is more complex due to the formation of micelles. At time zero, only the PEO peak was visible by ^1^H NMR (Fig. 5c) because micelle formation of the P(MAC-SC7A) segment resulted in signal suppression due to fast transverse relaxation of the proton signals. Over time, proton signals from degraded monomers and bis-HPA were observed although with slower formation kinetics. On day 55, a higher ratio of bis-HPA to MAC monomer was found. Quantitative analysis of the monomer and bis-HPA peaks (d3 + d4) showed a *t*_1/2_ of ~27 days at pH 7.4 (Fig. 5e).

### Safety evaluation of PSC7A NP over short and long term

**Fig. 6:**
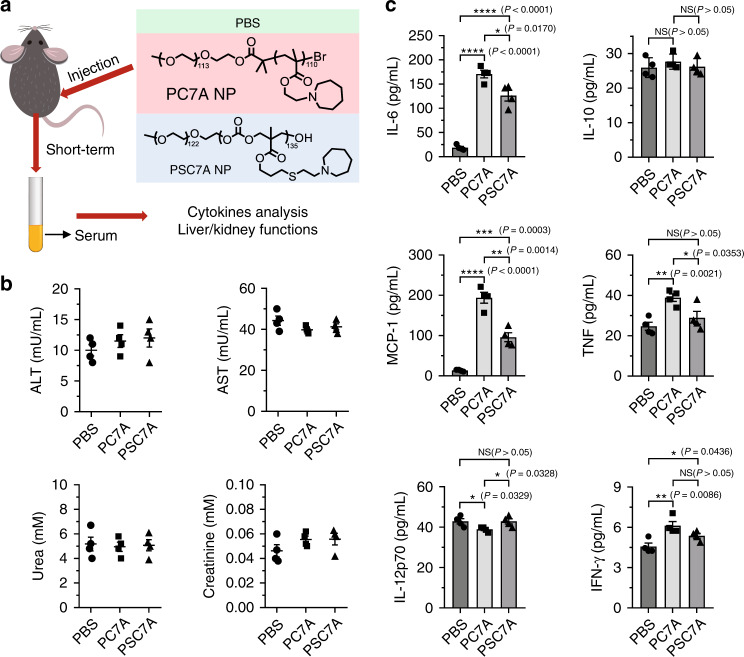
Short-term safety evaluation of degradable PSC7A NP and non-degradable PC7A NP. **a** C57BL/6 mice were subcutaneously injected with PBS, 300 μg PSC7A NP, or 300 μg PC7A NP on the right flank. **b** Serum concentrations of alanine aminotransferase (ALT), aspartate aminotransferase (AST), urea, and creatinine levels were quantitatively measured by Abcam^TM^ Assay Kit 24 h after injection. *n* = 4 biologically independent mouse samples in each group. Data are presented as means ± s.e.m. **c** Serum concentrations of interleukin-6 (IL-6), interleukin-10 (IL-10), monocyte chemoattractant protein-1 (MCP-1), interferon-*γ* (IFN-*γ*), tumor necrosis factor (TNF), and interleukin-12p70 (IL-12p70) protein levels were quantitatively measured by BD^TM^ CBA Mouse Inflammation Kit 24 h after injection. *n* = 4 biologically independent mouse samples in each group. Data are presented as means ± s.e.m. and statistical significance was calculated using Student’s two-tailed *t* test (^****^P < 0.0001, ^***^P < 0.001, ^**^P < 0.01, ^*^P < 0.05) and the exact *P* values are indicated in the figures.

A primary goal of converting the original UPS polymers into biodegradable dUPS polymers is to improve the safety profiles. Particularly for polymers that actively engage the innate immune response through STING pathway, the safety indication is paramount for repeated administration during therapy. In this study, we directly compared the PSC7A polymer with its non-degradable predecessor, PC7A (Fig. 6a). We injected C57BL/6 mice subcutaneously on their right flank with a high-dose of PSC7A NP or PC7A NP (300 μg, 10-fold of vaccine dose). Serum was collected 24 h following the injection, and systemic inflammatory cytokine concentrations were determined. No obvious acute kidney or liver toxicity was observed 24 h after treatment by either polymer (Fig. 6b). Generally, systemic cytokine expression was induced to a higher degree by PC7A NP than by PSC7A NP (Fig. 6c), indicating less systemic inflammatory response to the PSC7A NP. Histologic analysis of pivotal organs (heart, liver, spleen, kidney, and lung) are unremarkable after treatment by either polymer compared to PBS (Supplementary Fig. 18).

**Fig. 7:**
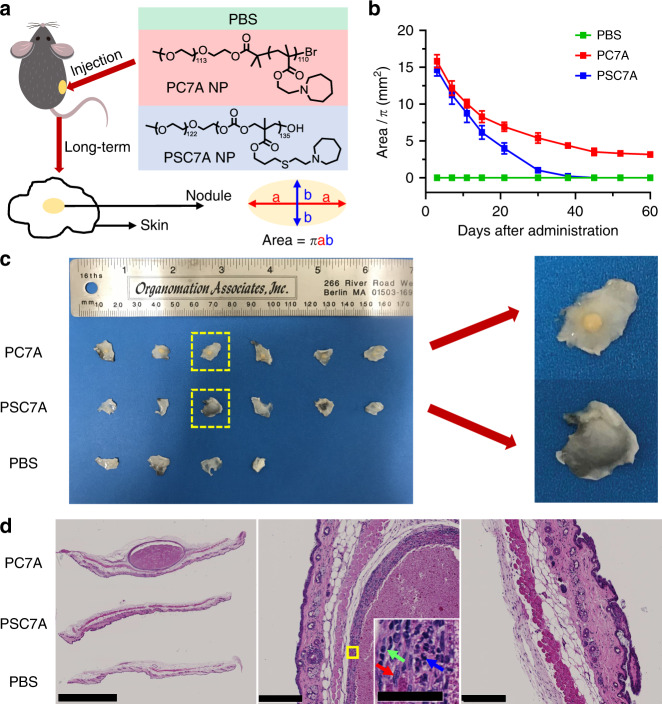
Long-term safety evaluation of degradable PSC7A NP and non-degradable PC7A NP. **a** C57BL/6 mice were subcutaneously injected with PBS, 300 μg PSC7A NP, or 300 μg PC7A NP on the right flank. Inflammatory nodules formed at the injection sites in PSC7A NP and PC7A NP groups. The surface areas of the nodules were calculated based on an ellipse model. **b** Change of nodule surface areas over time. *n* = 4 biologically independent mouse samples in PBS group, *n* = 6 biologically independent mouse samples in each PSC7A and PC7A group. Data are presented as means ± s.e.m. **c** Left: photographs of skin tissues taken from the injection sites on day 60, from top to bottom are mice treated with PC7A NP, PSC7A NP, and PBS. Right: magnification of skin tissues from PSC7A and PC7A groups. **d** Left: H&E staining of formalin-fixed, paraffin-embedded skin tissues (injection sites) on day 60, from top to bottom are mice treated with PC7A NP, PSC7A NP, and PBS. Scale bar: 2.5 mm. Middle: magnification of a nodule surrounded by granulomatous inflammation from PC7A group. Scale bar: 250 μm. Inset: magnification of the area marked by yellow square. The red, green, and blue arrows represent the macrophage, lymphocyte, and neutrophil, respectively. Scale bar: 50 μm. Right: magnification of skin tissue from PSC7A group. Scale bar: 250 μm. The whole set of experiment was repeated twice with similar results. In each experiment, two biologically independent mice were chosen randomly from each group on day 60. Skin tissues were harvested from each mouse and three adjacent sections of each sample were taken. One representative image from each group was shown.

The advantages of biodegradable PSC7A over non-degradable PC7A are more prominent in long-term safety studies. For this assay, mice were subcutaneously injected with PBS, 300 μg PSC7A NP, or 300 μg PC7A NP and observed over 60 days (Fig. 7a). The surface area of the resulting subcutaneous nodule was calculated based on an ellipse model to monitor progression (Fig. 7b). Within 1 day after administration, a large acute inflammatory reaction was observed at the injection site for both PC7A and PSC7A, likely due to innate immune stimulation. Histologically, we observed abundant neutrophilic infiltration and necrotic debris 24 h after injection (day 1 time point, Supplementary Fig. 19). Following this initial acute inflammatory reaction, the subcutaneous nodules reduced in size and gradually shifted into a chronic granulomatous inflammatory response, with more infiltration of macrophages and lymphocytes (day 15 and 30 time points, Supplementary Fig. 19). PSC7A-induced nodules reduced in size at a faster rate than those induced by PC7A, indicating the PSC7A polymer was degrading and being excreted from the injection site, allowing eventual healing of the tissue. The half-time of PSC7A nodule size reduction is about 13 days, consistent with degradation kinetics (Fig. 5). In contrast, the nodules induced by PC7A reduced in size until day 45 after administration, after which the nodules remained constant in size and appearance. On day 60, skin tissues at the injection site of all remaining mice were collected for histologic analysis (Fig. 7c). Grossly, 6/6 skin samples from the PC7A group contain a small, hard, yellow nodule. In contrast, none from the PSC7A group (0/6) contain nodules and resemble the PBS treated group in appearance. H&E staining on day 60 reveals nodules surrounded by granulomatous inflammation, with a “core/wall” appearance in mice treated with PC7A (Fig. 7d). Here, the “wall” is comprised mainly of macrophages with scattered lymphocytes and neutrophils, resulting from the acute and chronic inflammation and foreign body reaction. The “core” is necrotic in nature, consisting mainly of the proteinaceous debris of dying cells, with some infiltrating macrophages, neutrophils, and lymphocytes. In contrast, skin tissues from mice treated with PSC7A demonstrate complete disappearance of any nodules and restoration to a healthy state by comparison with those treated by PBS. Together, these data support complete degradation of PSC7A over time and a markedly improved safety profile compared to PC7A.

## Discussion

NPs for therapeutic delivery offer a unique opportunity to exploit molecular cooperativity to improve the precision of spatial targeting and temporal release. The UPS polymer library developed by our lab focuses on cooperative micelle disassembly in response to target tissue or organelle acidification. Previously, a UPS polymer has been utilized to delineate tumor margins based on metabolic acidosis of cancer cells undergoing aerobic glycolysis. Clear visualization of tumor margins was achieved, which led to significantly improved survival outcomes by image-guided surgery over white light surgery^18^. Recently, a ^64^Cu radionuclide-encoded UPS nanosensor was further developed leading to conspicuous detection of occult tumor nodules by positron emission tomography^38^. In these applications, tumor imaging relies on a single, low-dose administration of indocyanine green or radionuclide-conjugated NPs. No severe adverse side effects were found in animal studies and Phase I human patient trials^39^. For therapeutic applications, repeated administrations of the drug formulations may be necessary which can lead to rapid dose escalation of biopolymers. In these applications, design of degradable UPS polymers may prove essential to ensure safety and biocompatibility.

In this study, we successfully synthesized a library of degradable dUPS polymers which exhibit ultra-pH response as the original UPS polymers, but are capable of complete hydrolytic degradation within weeks after administration. The dUPS polymers made of polycarbonate backbone successfully rendered ultra-pH response from this study. Both PMMA and polycarbonate polymers rely on micellization to achieve cooperative deprotonation of tertiary amines, which occurs as high as 90% protonation degree. We noted an interesting difference in pH responsive behavior for polymers with dimethyl substituents of the tertiary amine (PSDMA or PDMA). The non-degradable PDMA polymer does not exhibit ultra-pH-sensitivity in the entire titration coordinate^31^. In contrast, PSDMA displays a biphasic pH response. When the protonation degree is above 50%, no micellization occurs and the pH response is broad; when the protonation degree decreases below 50%, micelle self-assembly is observed that coincides with dramatically sharpened pH response (Supplementary Fig. 10). These results suggest that the current polycarbonate backbone is slightly more hydrophobic than PMMA, which contribute to the micellization of PSDMA at protonation degree below 50%. These data further demonstrate phase transition is the driver for the cooperative pH response in this unique class of pH-sensitive nanomaterials.

Motivated by our previous finding of PC7A for cytosolic delivery of tumor antigens to lymph node-resident dendritic cells as well as STING activation for T-cell priming, we investigated the dUPS library for the immune adjuvant effect. Results show dUPS polymers with cyclic amines exhibit stronger STING-binding affinity and interferon induction than polymers with dialkyl amines, among which PSC7A is the most optimal. For PSC7A nanovaccine, the ultra-pH sensitive property is important in its pharmacologic properties, as the polymer remains in the micelle state for physical drainage and delivery to lymph node-resident antigen-presenting cells. Once endocytosed, the ultra-pH sensitivity then allows the NP to disassemble during early endosomal maturation, disrupting the endosome membranes and enabling the cytosolic delivery of PSC7A polymer for STING activation and of encapsulated antigen for presentation on MHC-I. Flow cytometry analysis in healthy mice shows that antigen-loaded PSC7A NP can increase the percentage of CD80^+^ dendritic cells and tetramer^+^ CD8^+^ T cells. In vivo studies show PSC7A nanovaccine can effectively induce robust antitumor immunity with increased long-term survival in two animal tumor models. Recent study shows PC7A polymer binds to a non-competing STING surface site distinct from the binding pocket of traditional small molecule agonists^40^. Polyvalent binding induces the formation of STING condensates, leading to a prolonged activation of downstream signaling pathways (TBK1, IRF3) for T-cell priming. We theorize PSC7A undergoes a similar biochemical mechanism of STING activation as the non-degradable PC7A analog.

The major advantage of dUPS over UPS polymers is in the safety and biodegradability of the degradable platform. The polycarbonate backbone is hydrolytically active, allowing the polymer to spontaneously degrade into biocompatible PEO segments and small molecules in aqueous environment. Degradation kinetics demonstrate that hydrolysis occurs at a slower rate at pH 7.4 than 6.5 (*t*_1/2_ is at 27 and 12 d, respectively), likely due to the micellization of the PSC7A segment restricting water access to the carbonate groups at pH 7.4. In vivo safety studies show both PSC7A and PC7A induce a rapid, innate inflammatory response in the short-term with less systemic cytokine level from PSC7A NP than PC7A NP. Long-term PSC7A degradation allows complete healing of the injection site while nodules surrounded by granulomatous inflammation persist in the PC7A site. These data support the improved safety and biocompatibility of the PSC7A polymers.

In summary, we report the synthesis of a new degradable series of ultra-pH sensitive (dUPS) copolymers based on a polycarbonate backbone with tunable pH transitions. pH titration experiments demonstrated superb buffer effect over a broad range of protonation degrees. Hydrophobic micellization was found to drive the ultra-pH sensitivity of the dUPS copolymers, indicating phase transition as a general strategy to render cooperative response in stimuli-responsive materials. Nanovaccines prepared by a physical mixture of PSC7A NPs and tumor antigens showed efficacious antitumor immunity with low systemic cytokine expression. The dUPS copolymers are hydrolytically active with pH-dependent degradation kinetics. Long-term safety evaluation demonstrated major advantages of degradable PSC7A in minimizing host response and foreign body reactions over non-degradable PC7A. The dUPS copolymers expand the existing arsenal of pH sensitive materials particularly in applications where repeated usage is necessary.

## Methods

### Materials

All reagents were purchased from commercial sources or synthesized and used without further purification unless specified. They were poly(ethylene glycol) methyl ether (mPEG_5k_-OH, *M*_n_ = 5.4 × 10^3^ g/mol measured by ^1^H NMR), 1-(3,5-bis-trifluoromethyl-phenyl)-3-cyclohexylthiourea (TU, synthesized)^41^, 1,8-diazabicyclo[5.4.0]undec-7-ene (DBU, >99%, Sigma-Aldrich), dipropylamine (DPA, 99%, Sigma-Aldrich), dibutylamine (DBA, >99.5%. Sigma-Aldrich), pyrrolidine (C5A, >99%, Sigma-Aldrich), piperidine (C6A, >99.5%), hexamethyleneimine (C7A, 99%, Sigma-Aldrich), ethylene sulfide (98%, Sigma-Aldrich), 2,2-dimethoxy-2-phenylacetophenone (DMPA, 99%, Sigma-Aldrich). 2-Dimethylaminoethanethiol hydrochloride (DMA-SH·HCl, 95%) and 2-diethylaminoethanethiol hydrochloride (DEA-SH·HCl, 95%) were purchased from Sigma-Aldrich. Other aminothiol hydrochloride molecules (Supplementary Fig. 1) were synthesized as reported^42^.

### Synthesis of PEO-*b*-PMAC copolymer

First, 5-methyl-5-allyloxycarbonyl-1,3-dioxan-2-one (MAC) monomer was synthesized as reported^43^. PEO-*b*-PMAC copolymer was synthesized by ring opening polymerization (ROP) with mPEG_5k_-OH as the initiator ([monomer]/[initiator]  = 200). Typically, a Schlenk reaction flask was charged with 0.4 g mPEG_5k_-OH, 3.2 g MAC monomer, and 16.0 mL dichloromethane (DCM) in a glove box filled with purified argon. After three freeze-pump-thaw cycles, 0.6 g TU and 0.16 mL DBU were introduced to start the polymerization. The reaction was placed in an oil bath at 30 °C for 15 h, then quenched by the addition of benzoic acid. The DCM solvent was removed by evaporation, and the concentrated residue was precipitated into an excess amount of cold ether. The purification process was repeated twice to remove any unreacted starting materials and impurity. The resulting PEO-*b*-PMAC copolymers were characterized by 400 MHz ^1^H NMR, gel permeation chromatography (GPC, Viscotech GPCmax, PLgel 5 *µ*m MIXED-D columns by Polymer Labs. THF with 1% *v*/*v* TEA was used as eluent at 1.0 mL/min). For PEG_123_-*b*-PMAC_125_, ^1^H NMR (400 MHz, CDCl_3_, 25 °C): *δ* 5.92-5.84 (m, 1H, -C*H* = CH_2_), 5.33-5.23 (m, 2H, -CH = C*H*_2_), 4.63 (br, 2H, -OC*H*_2_CH = CH_2_), 4.38-4.22 (m, 4H, -OC*H*_2_-C-C*H*_2_O-), 3.64 (s, 2H, -OC*H*_2_C*H*_2_O-), 3.38 (s, 3H, -OC*H*_3_), 1.28 (s, 3H, C*H*_3_), 1.22 (s, 3H, C(C*H*_3_)CH_2_OH). GPC (THF, IR): *M*_n_ = 2.58 × 10^4^ g/mol, *M*_w_ = 3.47 × 10^4^ g/mol, *M*_w_/*M*_n_ = 1.35).

### Synthesis of PEO-*b*-P(MAC-SR·HCl) (PSR) copolymers

PEO-*b*-P(MAC-SR·HCl) copolymer was synthesized by the thiol-ene reaction between allyl-containing PEO-*b*-PMAC and the aminothiol hydrochloride. Below we chose the synthesis of PEO-*b*-P(MAC-SDEA·HCl) as an example. First, 0.1 g PEO_123_-*b*-PMAC_125_ (0.419 mmol) was dissolved in 15 mL DMF in a quartz flask and stirred under nitrogen for 10–20 mins. Then 1.06 g DEA-SH·HCl (6.29 mmol) and 21.5 mg DMPA (0.084 mmol) were added into the flask. After nitrogen purge for additional 20 mins, the flask was put under a UV light (365 nm) to initiate the reaction. After 12 h, the reaction mixture was dialyzed in distilled water and lyophilized to obtain a white powder. The series of PEO-*b*-P(MAC-SR·HCl) copolymers were verified by ^1^H NMR and GPC. The results are shown in Supplementary Figs. 2–8 and summarized in Supplementary Table 1.

### Preparation of nanoparticles

PSC7A NP was used as an example. In a typical procedure, 10 mg PSC7A copolymer was dissolved in distilled water with 150 mM NaCl. NaOH solution was added to adjust the final pH value above 8.0. The excess NaOH and salts were removed by three cycles of ultracentrifugation using 3000 Da molecular weight cutoff centrifugal tube. Distilled water was added to the micelle solution to adjust the polymer concentration to 1.0 mg/mL.

### pH titration experiments

PSC7A was used as an example. In a typical experiment, 10 mg PSC7A copolymer was first dissolved in 10 mL distilled water to make the polymer concentration at 1.0 mg/mL. NaCl was added to adjust the salt concentration to 150 mM. Then NaOH solution was added to completely deprotonate the PSC7A copolymer. pH titration was carried out by adding small volumes (1 μL in increments) of 0.5 M HCl solution under stirring. The pH values were measured by a Mettler Toledo pH meter with a microelectrode. The pH decrease in the whole range was monitored as a function of total added HCl volume. The complete protonated state (100% protonation degree) and deprotonated state (0% protonation degree) were determined by the two extreme value points of the 1st derivation of pH titration curves. At specific protonation degrees, 100 μL of polymer solution was transferred into a specific cell and measured by DLS (Malvern Nano Zetasizer, He-Ne laser, *λ* = 633 nm). Other PSR copolymers followed the similar titration procedure.

### ^1^H NMR measurements

PSC7A copolymer in deuterated water (D_2_O) at protonation degrees of 0%, 20%, 40%, 60%, 80%, and 100% were obtained by adding different volumes of NaOD based on the pH titration coordinate. The solutions were measured by ^1^H NMR. The proton signal of PEO segment remained constant and was used as internal reference.

### Transmission electron microscopy measurements

PSC7A was used as an example. PSC7A-HCl polymer (1.0 mg/mL) was first dissolved in aqueous solution with 150 mM NaCl. Then, 0.5 M NaOH solution was added to deprotonate the PSC7A copolymer under stirring. To arrive at the desired protonation degree, we added appropriate amount of NaOH solution based on the pH titration data. For PSC7A, protonation degrees to 95% and 85% were achieved. Then the polymer solutions were diluted to 0.2 mg/mL. For TEM experiments, the solution was dropped on the 300-mesh carbon copper grid. After the grid was dried, distilled water was used to rinse the grid for several seconds to remove excess NaCl before the addition of phosphotungstic acid for negative staining. TEM images were obtained from Tecnai Spirit TEM. Similarly, PSDMA copolymer with the protonation degrees of 55% and 45% were imaged by TEM.

### Isothermal titration calorimetry

ITC was used to measure the binding affinities between STING dimer and dUPS copolymers using a Malvern ITC200 microcalorimeter. Titrations were performed at 20 °C in buffer containing 25 mM HEPES (pH 6.5) and 150 mM NaCl. The titration traces were integrated by NITPIC 1.2.7, and the curves were fitted by SEDPHAT 15.2b. The figures were prepared using GUSSI 1.4.2 (http://biophysics.swmed.edu/MBR/software.html).

### Cell culture

THP1-ISG cells were provided by Dr. Z. J. Chen (UT Southwestern). B16F10 cells were provided by Dr. Y. X. Fu (UT Southwestern). TC-1 cells were provided by Dr. T. C. Wu (John Hopkins University). All cells were cultured at 37°C in an atmosphere of 5% (v/v) CO_2_. TC-1 and B16F10 cells were cultured in Dulbecco’s modified Eagle’s medium (DMEM) supplemented with 10% (v/v) fetal bovine serum and 1% (v/v) penicillin–streptomycin antibiotics. THP1-ISG cells were cultured in RPMI 1640 supplemented with 10% (v/v) fetal bovine serum and 1% (v/v) penicillin-streptomycin antibiotics.

### STING reporter experiments

THP1-ISG cells (5 × 10^5^ cells/mL) were incubated with phorbol 12-myristate 13-acetate (PMA) in complete medium (RPMI-1640, 10% fetal bovine serum, 100 U/mL penicillin G sodium, and 100 μg/mL streptomycin) at 37 °C in 5% CO_2_ and normal O_2_ level for 48 h, and replenished with fresh medium for another 24 h. Then the cells were incubated with fresh medium with different dUPS copolymers (0.5 μM) for 48 h. The levels of IRF-induced Lucia luciferase in the cell culture supernatant were assessed with QUANTI-Luc™, a luciferase detection reagent.

### Immune cell profiling after vaccine treatment

The PSC7A nanovaccine was made by physically mixing E7 peptide and PSC7A NPs. Non-degradable PC7A based nanovaccine was used for comparison. Six- to eight-week-old C57BL/6 mice were injected subcutaneously at the tail base with PBS, E7p only (0.5 μg), PSC7A NP only (30 μg), PC7A nanovaccine (0.5 μg E7p in 30 μg PC7A NP), or PSC7A nanovaccine (0.5 μg E7p in 30 μg PSC7A NP) three times in 6 day intervals. At 24 h after the last administration (on day 13), the mice were sacrificed. Their inguinal lymph nodes were harvested, dispersed into single-cell suspensions, and stained with the indicated antibodies for flow cytometry analysis. The following primary antibodies were used for staining: anti-CD3-APC (Biolegend, cat. No. 100235, clone 17A2), anti-CD45-PerCP (Biolegend, cat. no. 103129, clone 30-F11), anti-CD4-FITC (Biolegend, cat. no. 100405, clone GK1.5), anti-CD8-AF700 (Biolegend, cat. no. 100729, clone 53-6.7), anti-H-2D^b^/HPV16 E7 (RAHYNIVTF) MHC Tetramer-PE (Immudex, cat. no. JA2195), anti-CD11c-FITC (Biolegend, cat. no. 117305, clone N418), and anti-CD80-PE/Cy7 (Biolegend, cat. no. 104733, clone 16-10A1). Flow data were acquired on a BD LSRFortessa™ Flow Cytometer and analyzed using FACSDiva 8.0.1 software.

### Animal experiments

All animal experiments were approved by the Institution Animal Care and Use Committees of The University of Texas Southwestern Medical Center and were consistent with local, state, and federal guidelines as applicable. Mice were housed in a barrier facility with a 12-h light/dark cycle and maintained on standard chow (2916 Teklad Global). The temperature range for the housing room is 68–79 °F (average is ~72 °F) and the humidity range is 30–50% (average is ~50%). Wildtype (WT) C57BL/6 mice were obtained from the UTSW Mouse Breeding Core Facility. STING knockout (STING-KO) C57BL/6 mice were provided by Dr. Y. X. Fu (UT Southwestern).

### PSC7A vaccine and tumor therapy experiments

The nanovaccine was made by physically mixing tumor specific antigenic peptides and PSC7A NPs. Non-degradable PC7A based nanovaccines were used for comparison. Six- to eight-week-old C57BL/6 mice were inoculated with 2 × 10^5^ TC-1 cells or B16F10 melanoma cells subcutaneously on their right thighs. In TC-1 tumor model, mice were injected subcutaneously into the tail base of PBS, E7p only (0.5 μg), PSC7A NP only (30 μg), low-dose PSC7A nanovaccine (0.1 μg E7p in 6 μg PSC7A NP), high-dose PSC7A nanovaccine (0.5 μg E7p in 30 μg PSC7A NP), or high-dose PC7A nanovaccine (0.5 μg E7p in 30 μg PC7A NP) on day 8, 14, and 24 after inoculation. In B16F10 tumor model, mice were injected subcutaneously into the tail base of PBS, Trp1,2 only (0.5 μg Trp1_214–237_ and 0.5 μg Trp2_173–196_), PSC7A NP only (30 μg), low-dose PSC7A nanovaccine (0.1 μg Trp1 and 0.1 μg Trp2 in 6 μg PSC7A NP), high-dose PSC7A nanovaccine (0.5 μg Trp1 and 0.5 μg Trp2 in 30 μg PSC7A NP), and high-dose PC7A nanovaccine (0.5 μg Trp1 and 0.5 μg Trp2 in 30 μg PC7A NP) on day 5, 10, and 15 after inoculation. Tumor growth was subsequently measured using a digital caliper and calculated as 0.5 × length × width^2^. Mice were sacrificed when the tumor volumes reached 2000 mm^3^ or the body weight loss exceeded 20%.

### STING-KO mice tumor therapy experiments

The nanovaccine was made by physically mixing tumor specific antigenic peptides and PSC7A NPs. Six- to eight-week-old WT and STING-KO C57BL/6 mice were inoculated with 2 × 10^5^ TC-1 cells subcutaneously on their right thighs. Either PBS or PSC7A vaccine (0.5 μg E7p in 30 μg PSC7A NP) was subcutaneously injected at the tail base on day 5, 10, and 15 after inoculation. Tumor growth was subsequently measured using a digital caliper and calculated as 0.5 × length × width^2^. Mice were sacrificed when the tumor volumes reached 2000 mm^3^ or the body weight loss exceeded 20%.

### Degradation studies of PSC7A in pH 6.5 and 7.4 buffer solutions

The deuterated phosphate buffer solutions at pH 6.5 and 7.4 were prepared by Na_2_HPO_4_ and NaH_2_PO_4_ in D_2_O (50 mM). NaCl was added to reach a final concentration at 150 mM. In pH 6.5 solution, 5.0 mg PSC7A copolymer was dissolved in 1.0 mL deuterated phosphate buffer solution to make the polymer concentration to 5.0 mg/mL. The pH of the polymer solution was further adjusted to 6.5 by concentrated NaOD and DCl solutions. The tube was then sealed and placed into the 37 °C shaker with a speed of 150 rpm. At certain times, the polymer solution was transferred to NMR tube for ^1^H NMR measurement. The pH of the polymer solution was adjusted every other day. The pH 7.4 solution study followed the similar procedure.

### H&E staining

Fresh tissues were first fixed in 10% formalin solution, and then were embedded in paraffin, sectioned and H&E stained by the Molecular Pathology Core at UTSW. The slides were scanned using Hamamatsu Nanozoomer 2.0HT and the images were analyzed using NDP.view 2.7.25 software.

### Statistics and reproducibility

TEM experiments of PSC7A polymer solutions at 85% and 95% protonation degrees and PSDMA polymer solutions at 45% and 55% protonation degrees were repeated three times independently with similar results, and one representative image from each group was shown. For histologic analysis of pivotal organs for short-term safety evaluation, the experiment was repeated twice with similar results. In each experiment, three biologically independent mice were chosen randomly from each group. A set of organs were harvested from each mouse and five adjacent sections of each set of samples were taken. One set of representative images from each group was shown. For histologic analysis of skin tissues for long-term safety evaluation, the experiment was repeated twice with similar results. In each experiment, two biologically independent mice were chosen randomly from each group at different time point. Skin tissues were harvested from each mouse and three adjacent sections of each sample were taken. One representative image from each group was shown.

### Statistical analysis

Statistical analysis was performed using Origin and Graphpad Prism. Statistical tests used are indicated in the figure legends. Data were considered statistically significant if *P* < 0.05 (^****^P < 0.0001, ^***^P < 0.001, ^**^P < 0.01, ^*^P < 0.05).

### Reporting summary

Further information on research design is available in the Nature Research Reporting Summary linked to this article.

## Supplementary information

Supplementary Information

Reporting Summary

## Data Availability

All the other data supporting the findings of this study are available within the article and its Supplementary Information files and from the corresponding author upon reasonable request. A reporting summary for this article is available as Supplementary Information.

## Source data

Source Data

## References

[1] Hu Y-B, Dammer EB, Ren R-J, Wang G (2015). The endosomal-lysosomal system: from acidification and cargo sorting to neurodegeneration. Transl. Neurodegener..

[2] Jensen PE (1990). Regulation of antigen presentation by acidic pH. J. Exp. Med..

[3] Young BP (2010). Phosphatidic acid is a pH biosensor that links membrane biogenesis to metabolism. Science.

[4] Estrella V (2013). Acidity generated by the tumor microenvironment drives local invasion. Cancer Res..

[5] Swietach P, Vaughan-Jones RD, Harris AL, Hulikova A (2014). The chemistry, physiology and pathology of pH in cancer. Philos. Trans. R. Soc. B Biol. Sci..

[6] Ling D (2014). Multifunctional tumor pH-sensitive self-assembled nanoparticles for bimodal imaging and treatment of resistant heterogeneous tumors. J. Am. Chem. Soc..

[7] Sonaje K (2010). Self-assembled pH-sensitive nanoparticles: a platform for oral delivery of protein drugs. Adv. Funct. Mater..

[8] Zhang X (2015). Tunable pH-responsive polymeric micelle for cancer treatment. ACS Macro Lett..

[9] Casey JR, Grinstein S, Orlowski J (2009). Sensors and regulators of intracellular pH. Nat. Rev. Mol. Cell Biol..

[10] Gerweck LE, Seetharaman K (1996). Cellular pH gradient in tumor versus normal tissue: potential exploitation for the treatment of cancer. Cancer Res..

[11] Li H-J (2016). Smart superstructures with ultrahigh pH-sensitivity for targeting acidic tumor microenvironment: instantaneous size switching and improved tumor penetration. ACS Nano.

[12] Duong HTT (2018). Smart vaccine delivery based on microneedle arrays decorated with ultra-pH-responsive copolymers for cancer immunotherapy. Biomaterials.

[13] Song C (2020). Long-circulating drug-dye-based micelles with ultrahigh pH-sensitivity for deep tumor penetration and superior chemo-photothermal therapy. Adv. Funct. Mater..

[14] Zhou K (2011). Tunable, ultrasensitive pH-responsive nanoparticles targeting specific endocytic organelles in living cells. Angew. Chem. Int. Ed..

[15] Zhou K (2012). Multicolored pH-tunable and activatable fluorescence nanoplatform responsive to physiologic pH stimuli. J. Am. Chem. Soc..

[16] Ma X (2014). Ultra-pH-sensitive nanoprobe library with broad pH tunability and fluorescence emissions. J. Am. Chem. Soc..

[17] Wang Y (2014). A nanoparticle-based strategy for the imaging of a broad range of tumours by nonlinear amplification of microenvironment signals. Nat. Mater..

[18] Zhao T (2016). A transistor-like pH nanoprobe for tumour detection and image-guided surgery. Nat. Biomed. Eng..

[19] Wang C (2015). A nanobuffer reporter library for fine-scale imaging and perturbation of endocytic organelles. Nat. Commun..

[20] Wang C (2017). Small-molecule TFEB pathway agonists that ameliorate metabolic syndrome in mice and extend C. elegans lifespan. Nat. Commun..

[21] Luo M (2017). A STING-activating nanovaccine for cancer immunotherapy. Nat. Nanotechnol..

[22] Chen W (2014). Advanced drug and gene delivery systems based on functional biodegradable polycarbonates and copolymers. J. Control. Release.

[23] Lonnecker AT, Lim YH, Wooley KL (2017). Functional polycarbonate of a D-Glucal-derived bicyclic carbonate via organocatalytic ring-opening polymerization. ACS Macro Lett..

[24] Mespouille L, Coulembier O, Kawalec M, Dove AP, Dubois P (2014). Implementation of metal-free ring-opening polymerization in the preparation of aliphatic polycarbonate materials. Prog. Polym. Sci..

[25] Rokicki G (2000). Aliphatic cyclic carbonates and spiroorthocarbonates as monomers. Prog. Polym. Sci..

[26] Zhu KJ, Hendren RW, Jensen K, Pitt CG (1991). Synthesis, properties, and biodegradation of poly(1,3-trimethylene carbonate). Macromolecules.

[27] Danquah M, Fujiwara T, Mahato RI (2010). Self-assembling methoxypoly(ethylene glycol)-b-poly(carbonate-co-L-lactide) block copolymers for drug delivery. Biomaterials.

[28] Chen X, McCarthy SP, Gross RA (1997). Synthesis, characterization, and epoxidation of an aliphatic polycarbonate from 2,2-(2-pentene-1,5-diyl)trimethylene carbonate (cHTC) ring-opening polymerization. Macromolecules.

[29] Brannigan RP, Dove AP (2017). Synthesis, properties and biomedical applications of hydrolytically degradable materials based on aliphatic polyesters and polycarbonates. Biomater. Sci..

[30] Feng J, Zhuo R-X, Zhang X-Z (2012). Construction of functional aliphatic polycarbonates for biomedical applications. Prog. Polym. Sci..

[31] Li Y (2016). Molecular basis of cooperativity in pH-triggered supramolecular self-assembly. Nat. Commun..

[32] Li Y (2016). Non-covalent interactions in controlling pH-responsive behaviors of self-assembled nanosystems. Polym. Chem..

[33] Barber GN (2015). STING: infection, inflammation and cancer. Nat. Rev. Immunol..

[34] Ishikawa H, Barber GN (2008). STING is an endoplasmic reticulum adaptor that facilitates innate immune signalling. Nature.

[35] Baccala R, Hoebe K, Kono DH, Beutler B, Theofilopoulos AN (2007). TLR-dependent and TLR-independent pathways of type I interferon induction in systemic autoimmunity. Nat. Med..

[36] Fuertes MB, Woo S-R, Burnett B, Fu Y-X, Gajewski TF (2013). Type I interferon response and innate immune sensing of cancer. Trends Immunol..

[37] Zitvogel L, Galluzzi L, Kepp O, Smyth MJ, Kroemer G (2015). Type I interferons in anticancer immunity. Nat. Rev. Immunol..

[38] Huang, G. et al. PET imaging of occult tumours by temporal integration of tumour-acidosis signals from pH-sensitive 64Cu-labelled polymers. *Nat. Biomed. Eng*. **4**, 314–324 (2019). 10.1038/s41551-019-0416-1 PMC6928453 31235828

[39] Voskuil FJ (2020). SHINE study group. Exploiting metabolic acidosis in solid cancers using a tumor-agnostic pH-activatable nanoprobe for fluorescence-guided surgery. Nat. Commun..

[40] Li, S. et al. A polyvalent STING agonist prolongs innate activation against cancer. *Nat. Biomed. Eng*., in press (2020).

[41] Natarajan A (2005). Synthetic studies toward aryl-(4-aryl-4H-[1,2,4]triazole-3-yl)-amine from 1,3-diarylthiourea as urea mimetics. J. Org. Chem..

[42] Hao J (2015). Rapid synthesis of a lipocationic polyester library via ring-opening polymerization of functional valerolactones for efficacious siRNA delivery. J. Am. Chem. Soc..

[43] Hu X, Chen X, Xie Z, Liu S, Jing X (2007). Synthesis and characterization of amphiphilic block copolymers with allyl side-groups. J. Polym. Sci. A Polym. Chem..

