# Supplementary material for: Polycarbonate-based ultra-pH sensitive nanoparticles improve therapeutic window
Source: Nat Commun. 2020 Nov 17;11:5828. doi: 10.1038/s41467-020-19651-7 (PMC7673035; doi:10.1038/s41467-020-19651-7)
Supplement: Supplementary file 1 — Supplementary Information [file 41467_2020_19651_MOESM1_ESM.pdf]

Supplementary Information

for

Polycarbonate-based Ultra-pH Sensitive Nanoparticles Improve Therapeutic Window

Xu Wang[1], Jonathan Wilhelm[1], Wei Li[1], Suxin Li[1], Zhaohui Wang[1], Gang Huang[1], Jian Wang[1], Houliang Tang[1], Sina Khorsandi[2], Zhichen Sun[1], Bret Evers[3] and Jinming Gao[1,*]

[1]Department of Pharmacology, Harold C. Simmons Comprehensive Cancer Center, University of Texas Southwestern Medical Center, Dallas, Texas 75390, United States. [2]Department of Radiology, University of Texas Southwestern Medical Center, Dallas, Texas 75390, United States. [3]Department of Pathology and Ophthalmology, University of Texas Southwestern Medical Center, Dallas, Texas 75390, United States.

[*]To whom correspondence should be addressed. E-mail: jinming.gao@utsouthwestern.edu

Supplementary Table 1. Characterization of dUPS polymers.

Copolymer	$M_{n,HNMR}$ (g·mol^{-1})	Number of repeating units of P(MAC-SR·HCl)	$M_{w,GPC}$ (g·mol^{-1})^c	$M_{n,GPC}$ (g·mol^{-1})^c	PDI^c
PEO_{123}-*b*-P(MAC-SDMA·HCl)$_{135}$	5.2 × 10^4	135^a	3.8 × 10^4	2.4 × 10^4	1.58
PEO_{123}-*b*-P(MAC-SDEA·HCl)$_{115}$	4.8 × 10^4	115^b	3.2 × 10^4	2.0 × 10^4	1.56
PEO_{123}-*b*-P(MAC-SDPA·HCl)$_{115}$	5.1 × 10^4	115^b	3.7 × 10^4	2.4 × 10^4	1.52
PEO_{123}-*b*-P(MAC-SDBA·HCl)$_{105}$	5.5 × 10^4	105^b	3.3 × 10^4	2.0 × 10^4	1.65
PEO_{123}-*b*-P(MAC-SC5A·HCl)$_{110}$	4.6 × 10^4	110^b	3.0 × 10^4	1.9 × 10^4	1.58
PEO_{123}-*b*-P(MAC-SC6A·HCl)$_{115}$	5.5 × 10^4	115^b	3.2 × 10^4	2.2 × 10^4	1.49
PEO_{123}-*b*-P(MAC-SC7A·HCl)$_{135}$	5.9 × 10^4	135^a	3.9 × 10^4	2.7 × 10^4	1.45

[a]The number of repeating units for the PEO-*b*-PMAC polymer precursor is 140.
[b]The number of repeating units for the PEO-*b*-PMAC polymer precursor is 125.
[c]$M_{w,GPC}$, $M_{n,GPC}$ and PDI ($M_{w,GPC}/M_{n,GPC}$) were obtained by using polystyrene as standard and THF (1% *v*/*v* TEA) as eluting solvent in GPC.

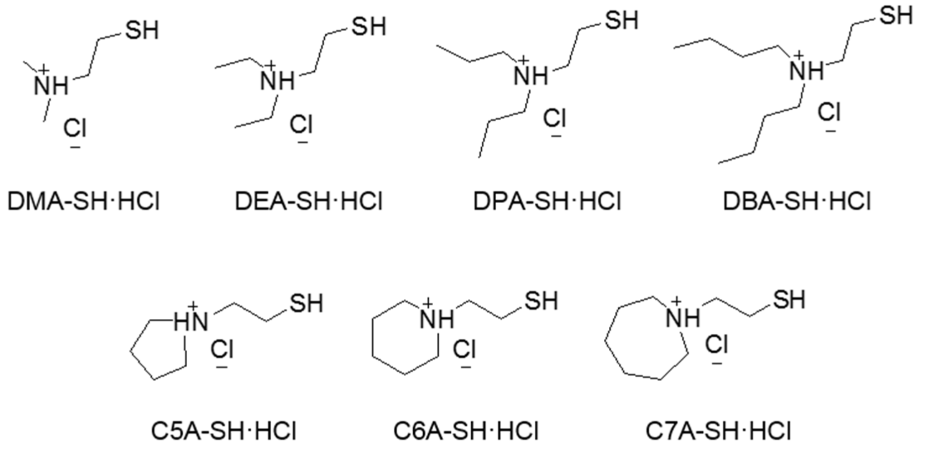

Supplementary Fig. 1 Chemical structures of amino thiol hydrochloride molecules.

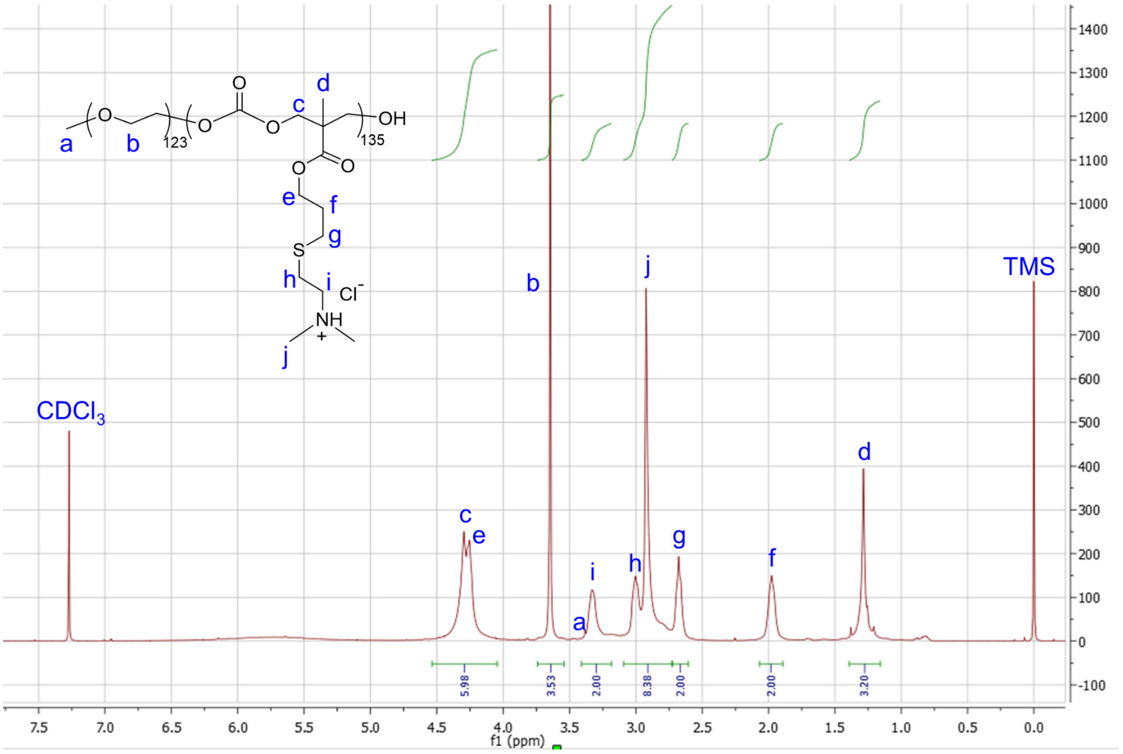

Supplementary Fig. 2 ^{1}H NMR spectrum of PEO$_{123}$-*b*-P(MAC-SDMA·HCl)$_{135}$ (PSDMA). δ 4.29 (s, 4H, -OCH_2-C-CH_2O-), 4.25 (t, 2H, -OCH_2CH$_2$CH$_2$S-), 3.64 (s, 4H, -OCH_2CH_2O-), 3.38 (s, 3H, -OCH_3), 3.35-3.31 (t, 2H, -SCH$_2$CH_2N-), 3.02-2.98 (m, 2H, -SCH_2CH$_2$N-), 2.92 (s, 6H, -N(CH_3)$_2$), 2.66 (t, 2H, -OCH$_2$CH$_2$CH_2S-), 2.00-1.95 (m, 2H, -OCH$_2$CH_2CH$_2$S-), 1.28 (s, 3H, -CH_3). Solvent: CDCl$_3$.

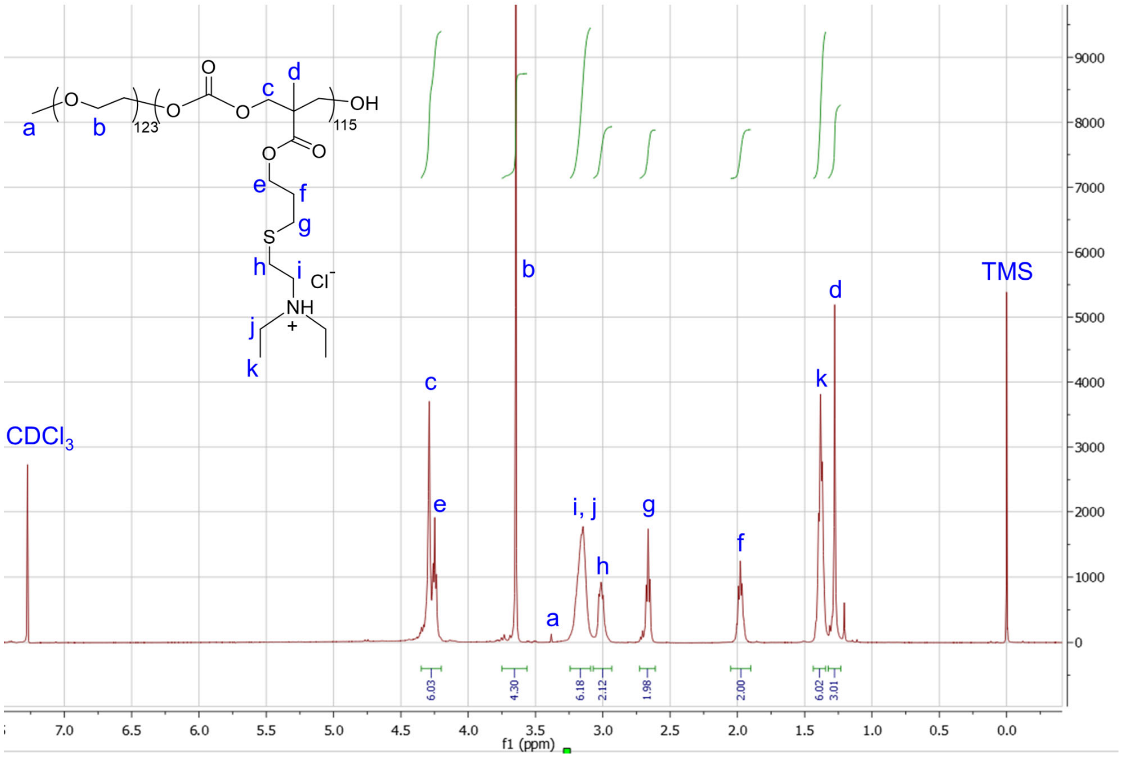

Supplementary Fig. 3 ^{1}H NMR spectrum of PEO$_{123}$-*b*-P(MAC-SDEA·HCl)$_{115}$ (PSDEA). δ 4.29 (s, 4H, -OCH_2-C-CH_2O-), 4.25 (t, 2H, -OCH_2CH$_2$CH$_2$S-), 3.64 (s, 4H, -OCH_2CH_2O-), 3.38 (s, 3H, -OCH_3), 3.20-3.14 (m, 6H, -SCH$_2$CH_2N(CH_2CH$_3$)$_2$-), 3.03-3.00 (m, 2H, -SCH_2CH$_2$N-), 2.66 (t, 2H, -OCH$_2$CH$_2$CH_2S-), 2.00-1.95 (m, 2H, -OCH$_2$CH_2CH$_2$S-), 1.38 (t, 6H, -N(CH$_2$CH_3)$_2$), 1.28 (s, 3H, -CH_3). Solvent: CDCl$_3$.

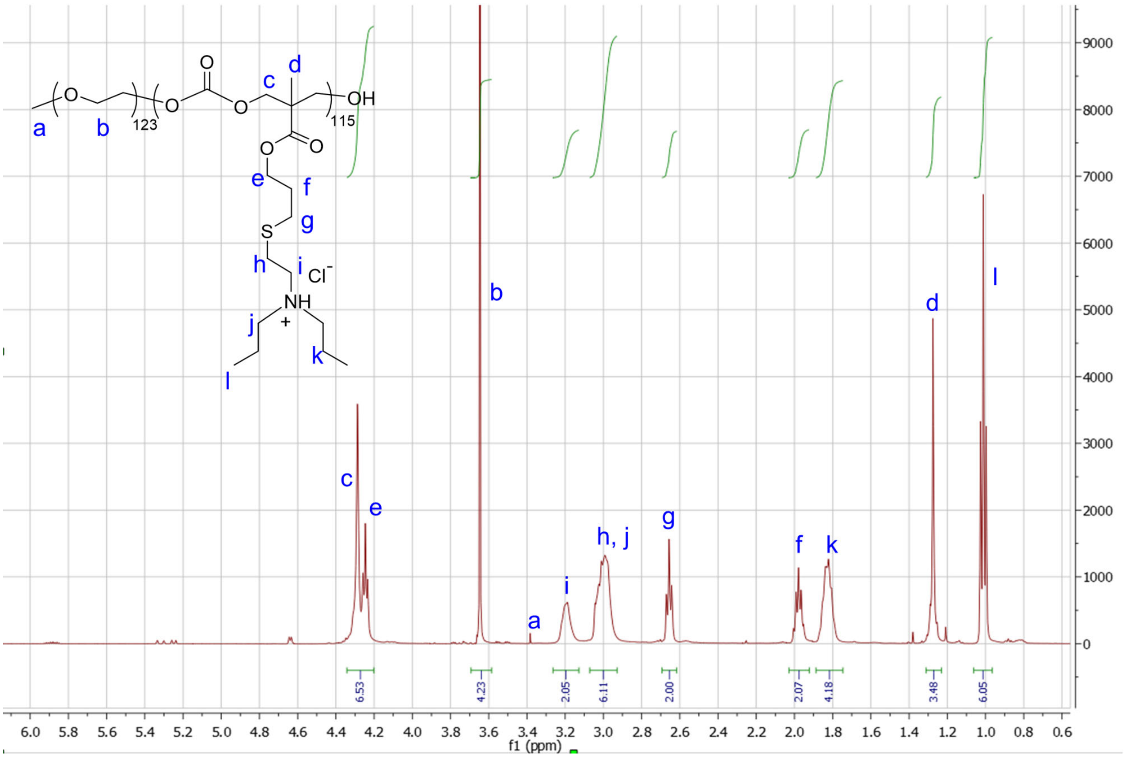

Supplementary Fig. 4 ^{1}H NMR spectrum of PEO$_{123}$-*b*-P(MAC-SDPA·HCl)$_{115}$ (PSDPA). δ 4.29 (s, 4H, -OC*H*$_2$-C-C*H*$_2$O-), 4.25 (t, 2H, -OC*H*$_2$CH$_2$CH$_2$S-), 3.64 (s, 4H, -OC*H*$_2$C*H*$_2$O-), 3.38 (s, 3H, -OC*H*$_3$), 3.19 (s, 2H, -SCH$_2$C*H*$_2$N-), 3.04-2.98 (m, 2H, -SC*H*$_2$CH$_2$N- and -N(C*H*$_2$CH$_2$CH$_3$)$_2$), 2.66 (t, 2H, -OCH$_2$CH$_2$C*H*$_2$S-), 2.00-1.95 (m, 2H, -OCH$_2$C*H*$_2$CH$_2$S-), 1.85-1.81 (m, 4H, -N(CH$_2$C*H*$_2$CH$_3$)$_2$), 1.28 (s, 3H, -C*H*$_3$), 1.01 (t, 6H, -N(CH$_2$CH$_2$C*H*$_3$)$_2$). Solvent: CDCl$_3$.

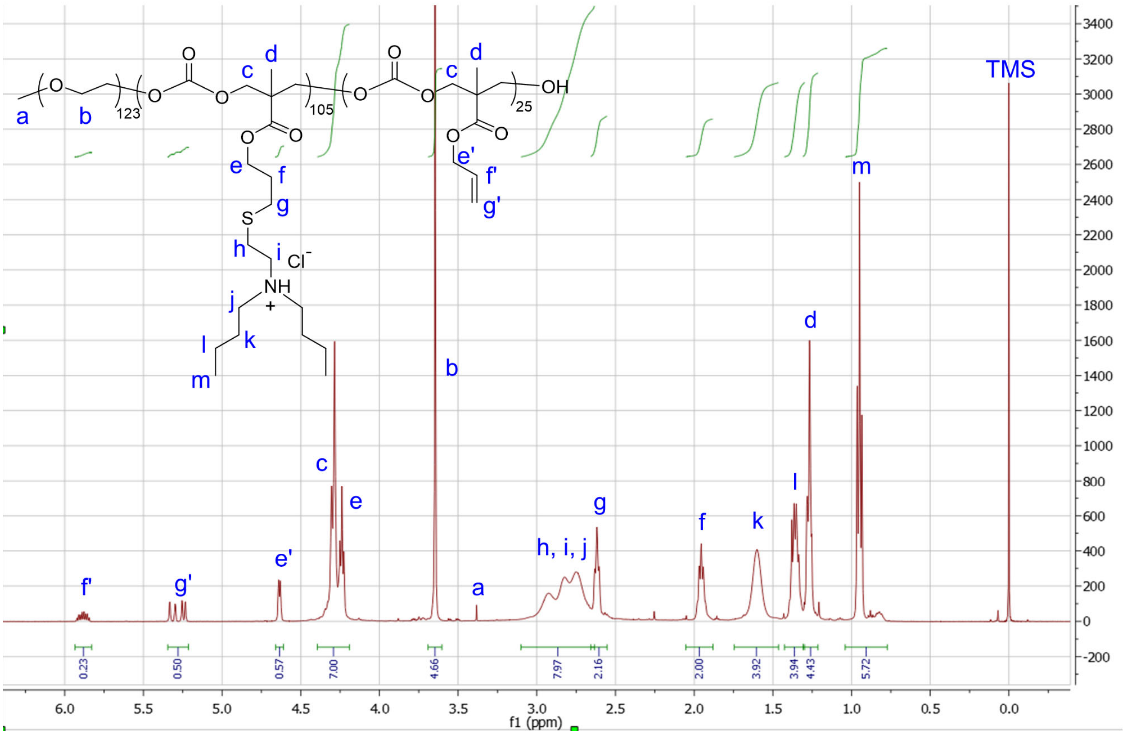

Supplementary Fig. 5 ^{1}H NMR spectrum of PEO_{123}-*b*-P(MAC-SDBA·HCl)$_{105}$ (PSDBA). δ 5.92-5.84 (m, 1H, -C*H*=CH_2), 5.33-5.23 (m, 2H, -CH=CH_2), 4.64-4.63 (d, 2H, -OCH_2CH=CH_2), 4.34-4.28 (m, 4H, -OCH_2-C-CH_2O-), 4.24 (t, 2H, -OC$H_2$$CH_2$$CH_2$S-), 3.64 (s, 4H, -OC$H_2CH_2$O-), 3.38 (s, 3H, -OC$H_3$), 2.92 (m, 2H, -SC$H_2$$CH_2$N-), 2.82 (m, 2H, -S$CH_2CH_2$N-), 2.75 (m, 4H, -N(C$H_2$$CH_2$$CH_2$$CH_3$)$_2$), 2.62 (t, 2H, -O$CH_2$$CH_2CH_2$S-), 1.98-1.93 (m, 2H, -OCH_2C$H_2$$CH_2$S-), 1.60 (s, 4H, -N($CH_2CH_2$$CH_2$$CH_3$)$_2$), 1.39-1.33 (m, 4H, -N($CH_2$$CH_2CH_2$$CH_3$)$_2$), 1.29-1.29 (m, 6H, -C$H_3$), 0.95 (t, 6H, -N($CH_2$$CH_2$$CH_2CH_3$)$_2$). Solvent: $CDCl_3$. A small percentage of allyl group on PMAC did not fully react with DBA-SH·HCl due to the steric hindrance of the dibutyl groups.

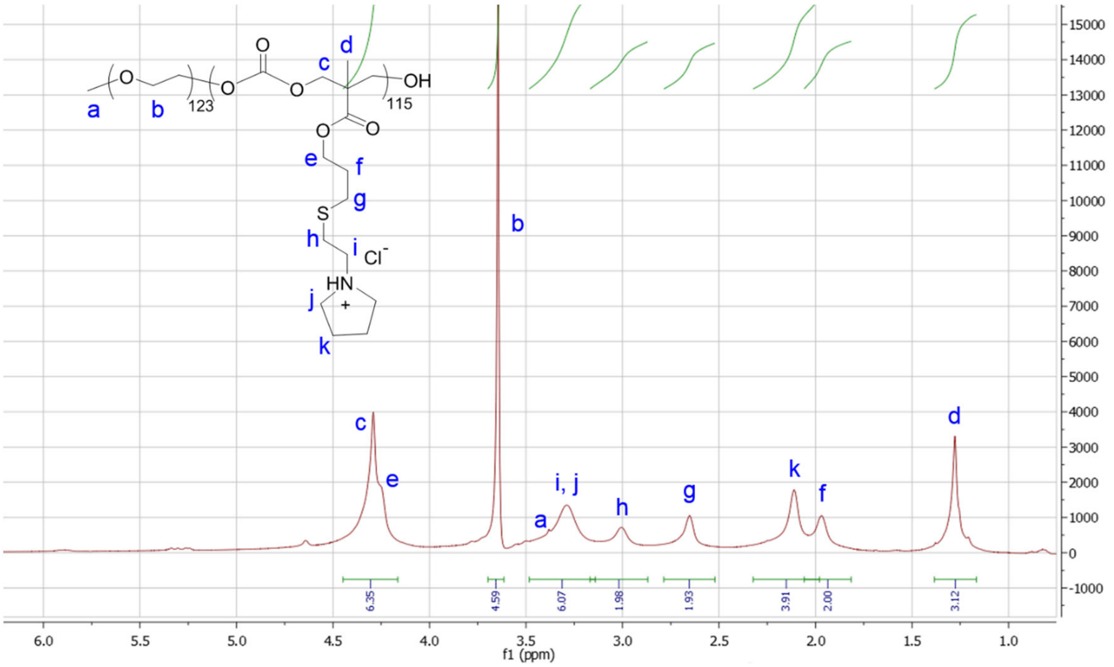

Supplementary Fig. 6 ^{1}H NMR spectrum of PEO$_{123}$-*b*-P(MAC-SC5A·HCl)$_{110}$ (PSC5A). *δ* 4.29 (s, 4H, -OC*H*$_2$-C-C*H*$_2$O-), 4.25 (t, 2H, -OC*H*$_2$CH$_2$CH$_2$S-), 3.64 (s, 4H, -OC*H*$_2$C*H*$_2$O-), 3.38 (s, 3H, -OC*H*$_3$), 3.55-3.29 (m, 6H, -SCH$_2$C*H*$_2$N(C*H*$_2$CH$_2$)$_2$), 3.01 (m, 2H, -SC*H*$_2$CH$_2$N-), 2.66 (t, 2H, -OCH$_2$CH$_2$C*H*$_2$S-), 2.11 (s, 4H, -N(CH$_2$C*H*$_2$)$_2$), 2.00-1.95 (m, 2H, -OCH$_2$C*H*$_2$CH$_2$S-), 1.28 (s, 3H, -C*H*$_3$). Solvent: CDCl$_3$.

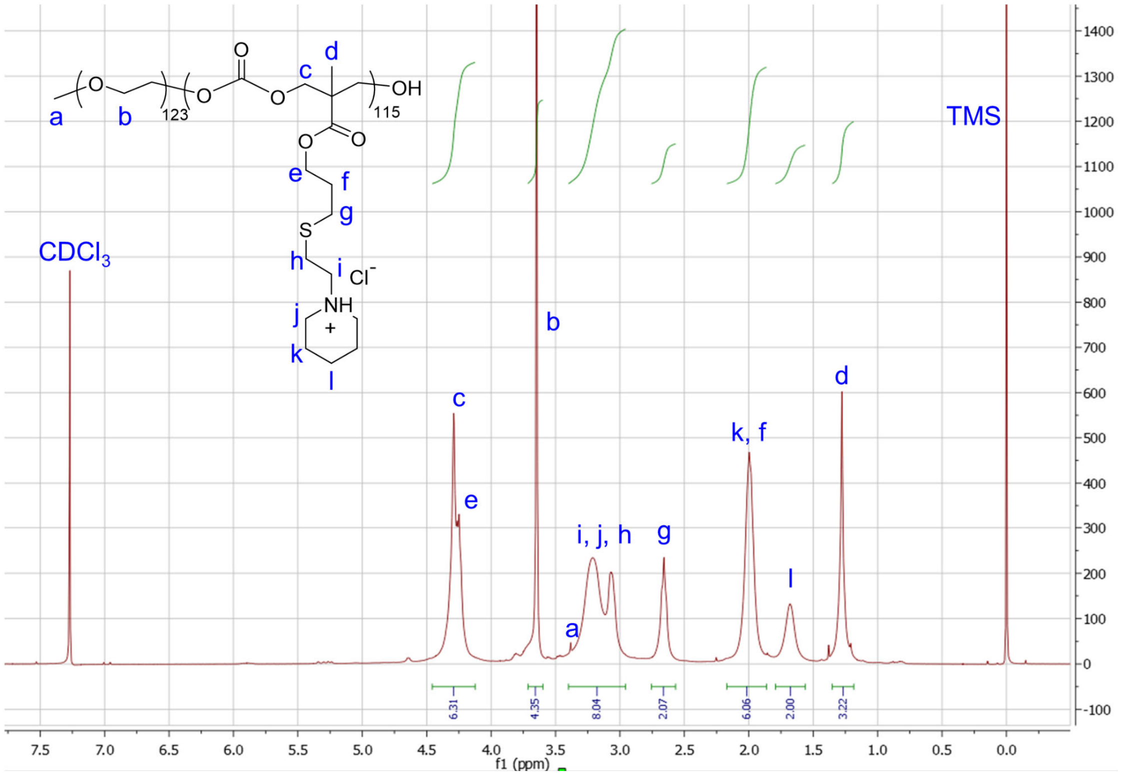

Supplementary Fig. 7 ^{1}H NMR spectrum of PEO$_{123}$-*b*-P(MAC-SC6A·HCl)$_{115}$ (PSC6A). δ 4.29 (s, 4H, -OCH_2-C-CH_2O-), 4.25 (t, 2H, -OCH_2CH$_2$CH$_2$S-), 3.64 (s, 4H, -OCH_2CH_2O-), 3.38 (s, 3H, -OCH_3), 3.21 (br, 6H, -SCH$_2$CH_2N(CH_2CH$_2$)$_2$CH$_2$), 3.07 (s, 2H, -SCH_2CH$_2$N-), 2.66 (t, 2H, -OCH$_2$CH$_2$CH_2S-), 2.00 (m, 6H, -OCH$_2$CH_2CH$_2$S- and -N(CH$_2$CH_2)$_2$CH$_2$), 1.68 (m, 2H, -N(CH$_2$CH$_2$)$_2$CH_2), 1.28 (s, 3H, -CH_3). Solvent: CDCl$_3$.

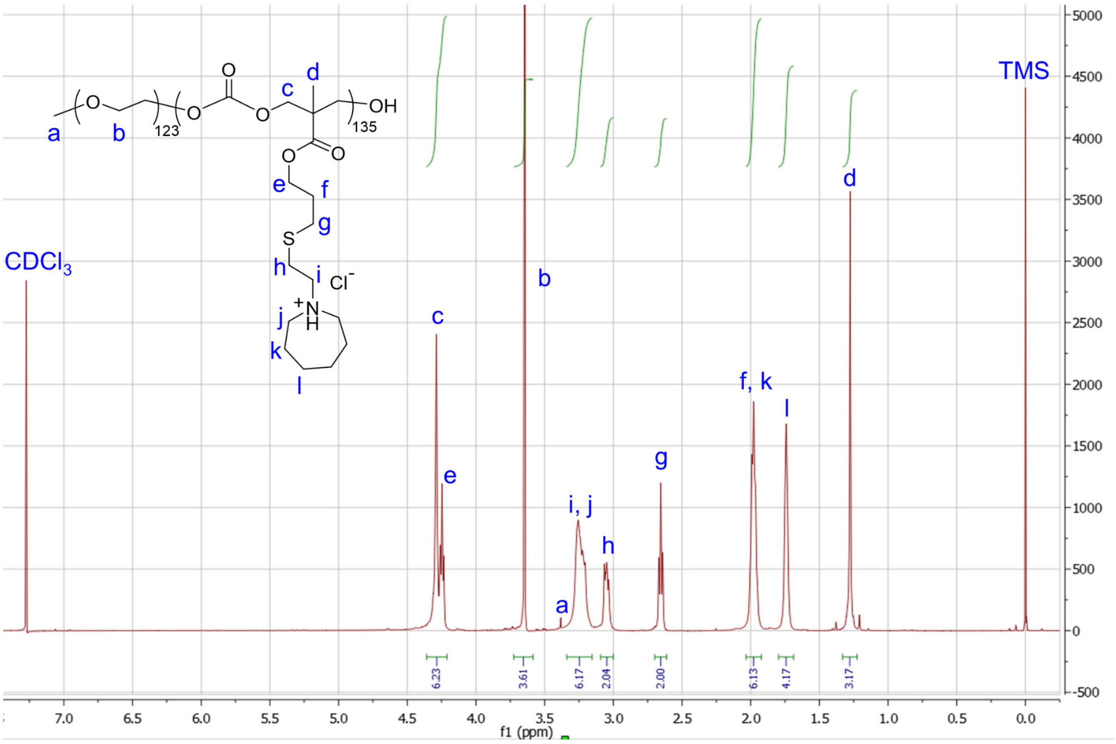

Supplementary Fig. 8 ^{1}H NMR spectrum of PEO$_{123}$-*b*-P(MAC-SC7A·HCl)$_{135}$ (PSC7A). δ 4.29 (s, 4H, -OCH_2-C-CH_2O-), 4.25 (t, 2H, -OCH_2CH$_2$CH$_2$S-), 3.64 (s, 4H, -OCH_2CH_2O-), 3.38 (s, 3H, -OCH_3), 3.26 (s, 2H, -SCH$_2$CH_2N-), 3.23-3.21 (m, 6H, -N(CH_2CH$_2$CH$_2$)$_2$), 2.66 (t, 2H, -OCH$_2$CH$_2$CH_2S-), 2.00-1.95 (m, 6H, -OCH$_2$CH_2CH$_2$S- and -N(CH$_2$CH_2CH$_2$)$_2$), 1.74 (s, 4H, -N(CH$_2$CH$_2$CH_2)$_2$), 1.28 (s, 3H, -CH_3). Solvent: CDCl$_3$.

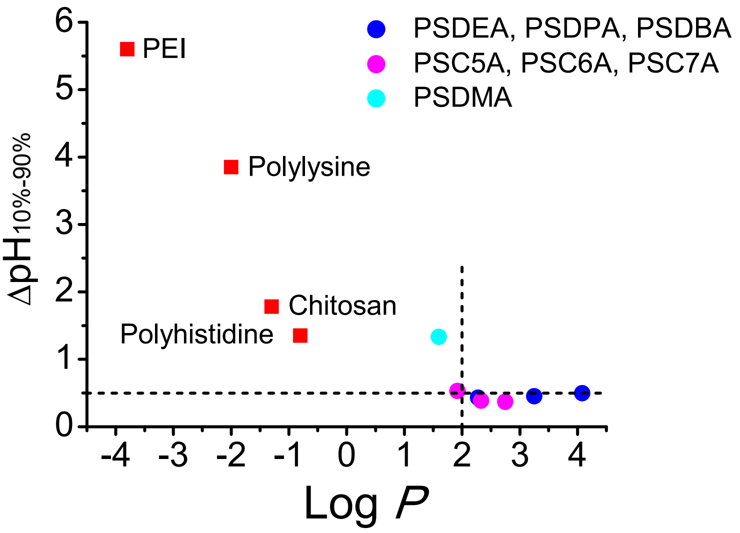

Supplementary Fig. 9 pH transition sharpness ($\Delta pH_{10\%-90\%}$) as a function of log *P* of dUPS repeating unit. Commonly used polybases (poly(ethyleneimine), chitosan, polyhistidine, polylysine) are shown for comparison.

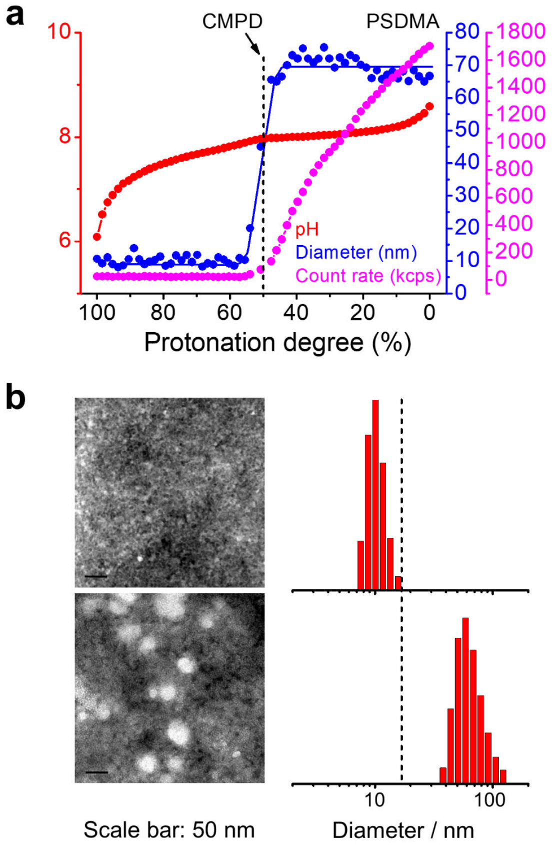

Supplementary Fig. 10 a Number-weighted hydrodynamic diameters and light scattering count rates as a function of protonation degree during the pH titration of PSDMA copolymer. PSDMA displays a divergent pH response. When the protonation degree is above 50%, no micellization occurs and the pH response is broad; when the protonation degree decreases below 50%, micelle self-assembly is observed that coincides with dramatically sharpened pH response. **b** TEM images and corresponding number-weighted hydrodynamic diameter distributions by dynamic light scattering (DLS) analysis of PSDMA in 150 mM NaCl solution at 55% and 45% protonation degrees, above and below CMPD (~50%). Scale bar: 50 nm. TEM measurements of PSDMA polymer solutions at 45% and 55% protonation degrees were repeated 3 times independently with similar results, and one representative image from each group was shown.

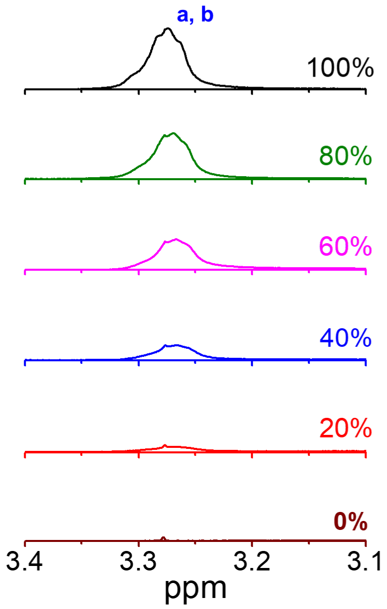

PSC7A

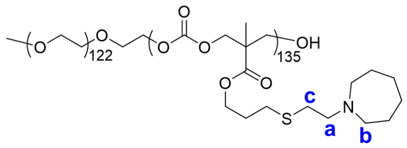

Chemical shift			Integration		
	a, b	c		a, b	c
0%	-	-	0%	-	-
20%	3.31-3.26	2.84-2.80	20%	132.8	56.9
40%	3.31-3.26	2.84-2.80	40%	299.6	113.4
60%	3.31-3.26	2.85-2.80	60%	498.8	172.5
80%	3.31-3.26	2.85-2.80	80%	683.5	228.8
100%	3.32-3.27	2.86-2.80	100%	846.6	273.2

Supplementary Fig. 11 ^{1}H NMR spectra in D_2O of methylene protons of PSC7A adjacent to the nitrogen atom at different protonation degrees (spectra of methylene protons adjacent to the sulfur atom (proton "**c**") were not shown); chemical shifts and peak integrations of different proton signals of PSC7A.

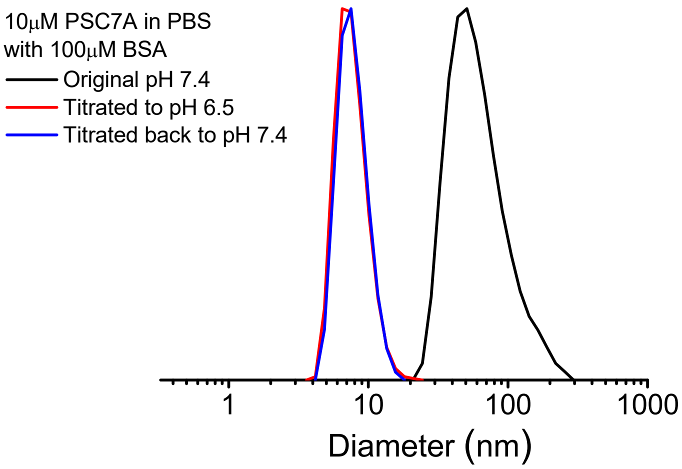

Supplementary Fig. 12 PSC7A PBS solution (10 μM) containing 100 μM bovine serum albumin (BSA) was adjusted to pH 7.4 and measured by DLS. The average number-weighted hydrodynamic diameter was ~45 nm, indicating micelle structure. Then the solution was titrated to pH 6.5 by adding a small amount of HCl and the polymer chains dissembled into unimers. In the presence of BSA, PSC7A polymer chains remained in the unimer status when it was titrated back from pH 6.5 to pH 7.4 as indicated (~7 nm).

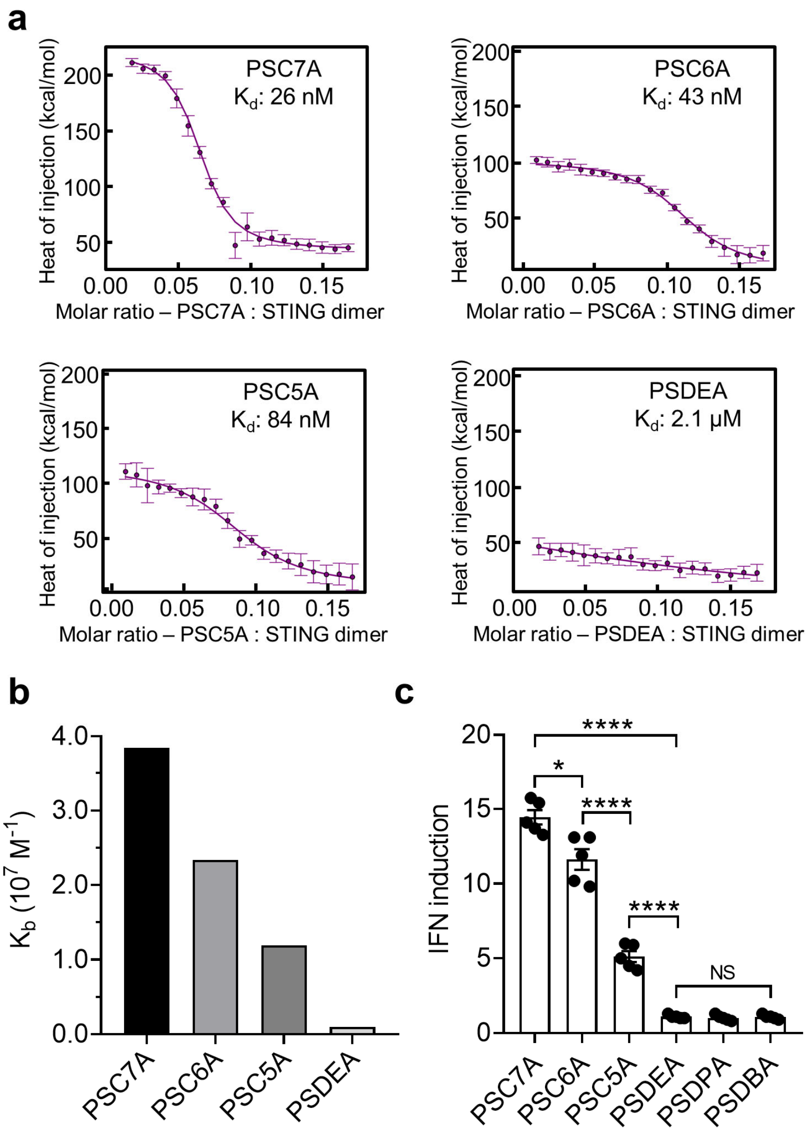

Supplementary Fig. 13 a Isothermal titration calorimetry (ITC) results of dUPS polymers. K_d: apparent dissociation constant. **b** Summary of K_b values (binding affinity, $K_b = 1/K_d$) of different dUPS polymers to STING from ITC experiments. **c** Interferon (IFN) induction levels of THP1-ISG cells incubated with dUPS copolymers (0.5 μM for 48h) correlate with STING binding affinity. n = 5 experimental replicates in each group. Data are presented as means ± s.e.m. and statistical significance was calculated using Student's two-tailed *t*-test (PSC7A *vs*. PSC6A: $^{*}P = 0.0105$, PSC7A *vs*. PSDEA: $^{****}P < 0.0001$, PSC6A *vs*. PSC5A: $^{****}P < 0.0001$, PSC5A *vs*. PSDEA: $^{****}P < 0.0001$.).

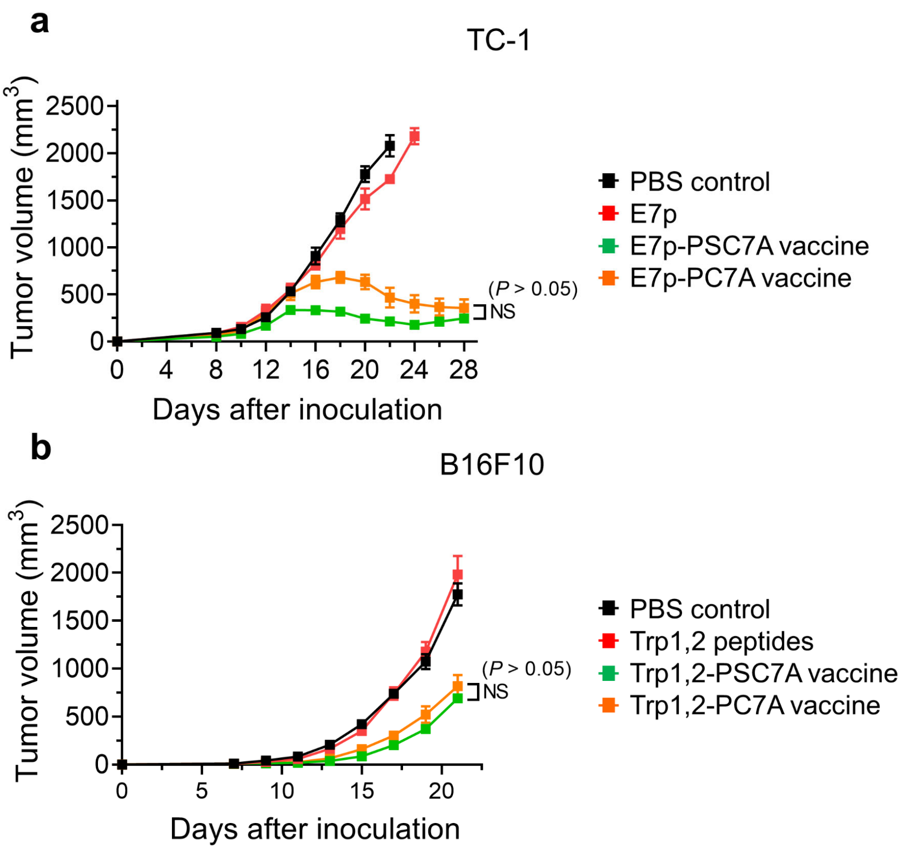

Supplementary Fig. 14 Comparison of PSC7A nanovaccine and PC7A nanovaccine antitumor efficacy in two animal tumor models. C57BL/6 mice inoculated with 2×10^5 **a** TC-1 or **b** B16-F10 melanoma cells were treated with PBS, tumor antigenic peptides, PSC7A vaccine, or PC7A vaccine at specific time points. TC-1 model: n = 7 biologically independent mice in each group. Tumor growth data are presented as means ± s.e.m. and statistical significance was calculated using two-way ANOVA with Holm-Sidak's multiple comparison testing (PSC7A vaccine *vs*. PC7A vaccine: not significant, based on 28d data after inoculation). B16F10 model: n = 7 biologically independent mice in each PBS, peptides only and PC7A vaccine group, n = 8 biologically independent mice in PSC7A vaccine group. Tumor growth data are presented as means ± s.e.m. and statistical significance was calculated using two-way ANOVA with Dunnett's multiple comparison testing (PSC7A vaccine *vs*. PC7A vaccine: not significant, based on 21d data after inoculation).

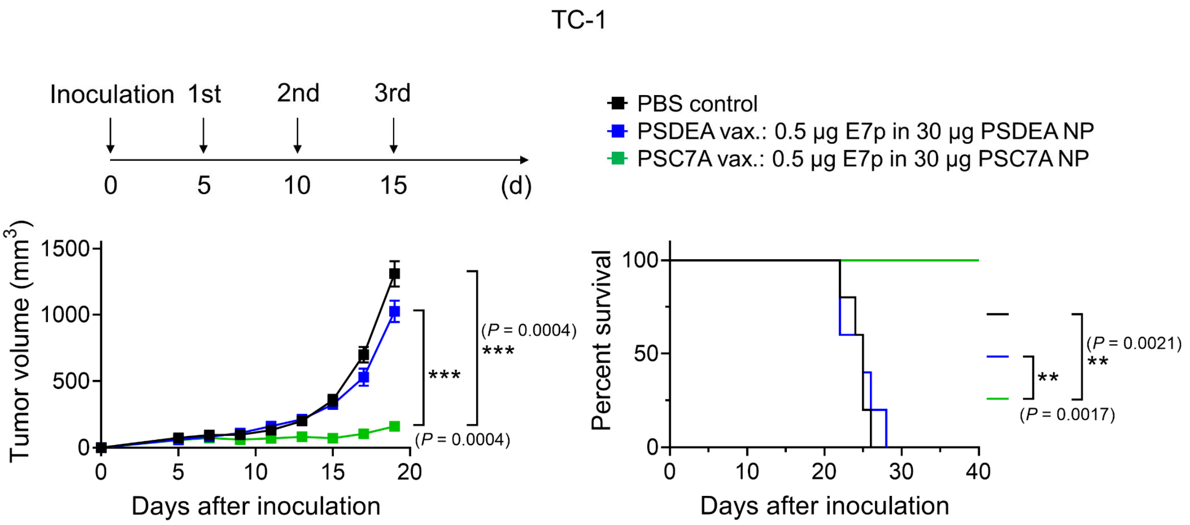

Supplementary Fig. 15 Comparison of PSC7A and PSDEA vaccines in tumor growth inhibition and animal survival. C57BL/6 mice inoculated with 2×10^5 TC-1 cells were treated with PBS, PSDEA vaccine and PSC7A vaccine at the tail base at specific time points after inoculation. The PSDEA vaccine failed to induce an efficacious anti-tumor immune response compared to control treated mice, while the PSC7A vaccine inhibited tumor growth and significantly prolonged survival. n = 5 biologically independent mice in each group. Tumor growth data are presented as means ± s.e.m. and statistical significance was calculated using two-way ANOVA with Tukey's multiple comparison testing (PSC7A vax. *vs*. PBS control: $^{***}P = 0.0004$, PSC7A vax. *vs*. PSDEA vax.: $^{***}P = 0.0004$, based on 19d data after inoculation). For survival analysis, the statistical significance was calculated by the log-rank test (PSC7A vax. *vs*. PBS control: $^{**}P = 0.0021$, PSC7A vax. *vs*. PSDEA vax.: $^{**}P = 0.0017$).

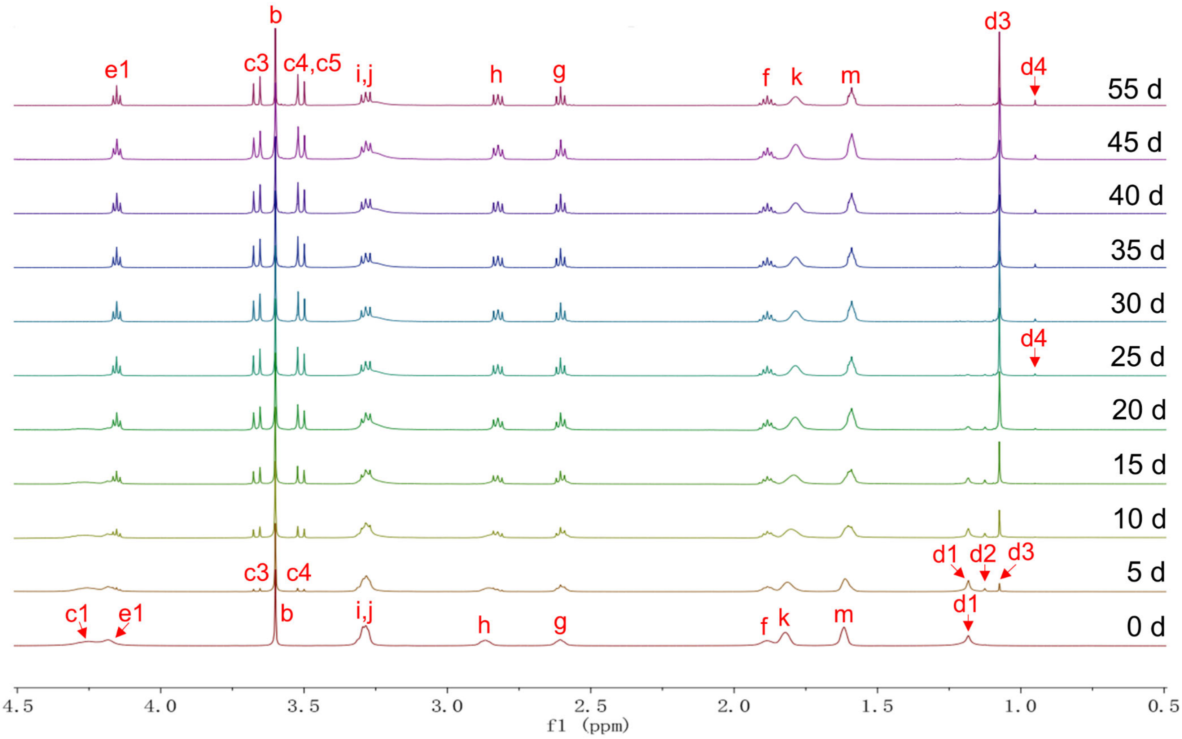

Supplementary Fig. 16 Complete ^{1}H NMR spectra of PSC7A copolymer in pH 6.5 deuterated buffer solution over 55 days. δ 4.25 (4H, -OCH_2-C-CH_2O-), 4.18-4.12 (2H, -OCH_2CH$_2$CH$_2$S-), 3.60 (4H, -OCH_2CH_2O-), 3.70-3.63 and 3.54-3.47 (4H, OH-CH_2-C-CH_2-OH), 3.33-3.17 (6H, -SCH$_2$CH_2N- and -N(CH_2CH$_2$CH$_2$)$_2$), 2.91-2.79 (2H, -SCH_2CH$_2$N-), 2.64-2.56 (-OCH$_2$CH$_2$CH_2S-), 1.93-1.84 (2H, -OCH$_2$CH_2CH$_2$S-), 1.84-1.73 (4H, -N(CH$_2$CH_2CH$_2$)$_2$), 1.65-1.55 (4H, -N(CH$_2$CH$_2$CH_2)$_2$), 1.18 (3H, -CH_3), 1.13 (3H, -CH_3), 1.07 (3H, -CH_3), 0.95 (3H, -CH_3).

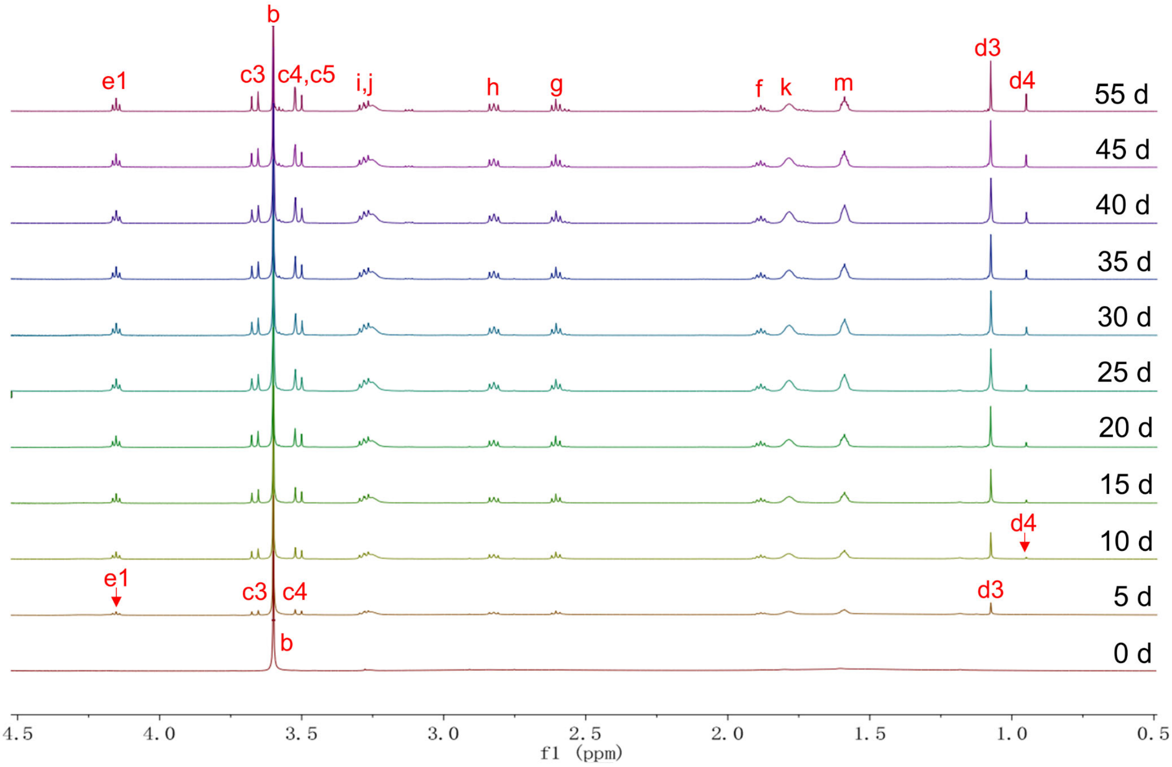

Supplementary Fig. 17 Complete ^{1}H NMR spectra of PSC7A copolymer in pH 7.4 deuterated buffer solution over 55 days. Complete ^{1}H NMR spectra of PSC7A copolymer in pH 6.5 deuterated buffer solution over 55 days. 4.18-4.12 (2H, -OCH_2CH$_2$CH$_2$S-), 3.60 (4H, -OCH_2CH_2O-), 3.70-3.63 and 3.54-3.47 (4H, OH-CH_2-C-CH_2-OH), 3.33-3.19 (6H, -SCH$_2$CH_2N- and -N(CH_2CH$_2$CH$_2$)$_2$), 2.86-2.79 (2H, -SCH_2CH$_2$N-), 2.64-2.56 (-OCH$_2$CH$_2$CH_2S-), 1.93-1.84 (2H, -OCH$_2$CH_2CH$_2$S-), 1.84-1.73 (4H, -N(CH$_2$CH_2CH$_2$)$_2$), 1.65-1.55 (4H, -N(CH$_2$CH$_2$CH_2)$_2$), 1.07 (3H, -CH_3), 0.95 (3H, -CH_3).

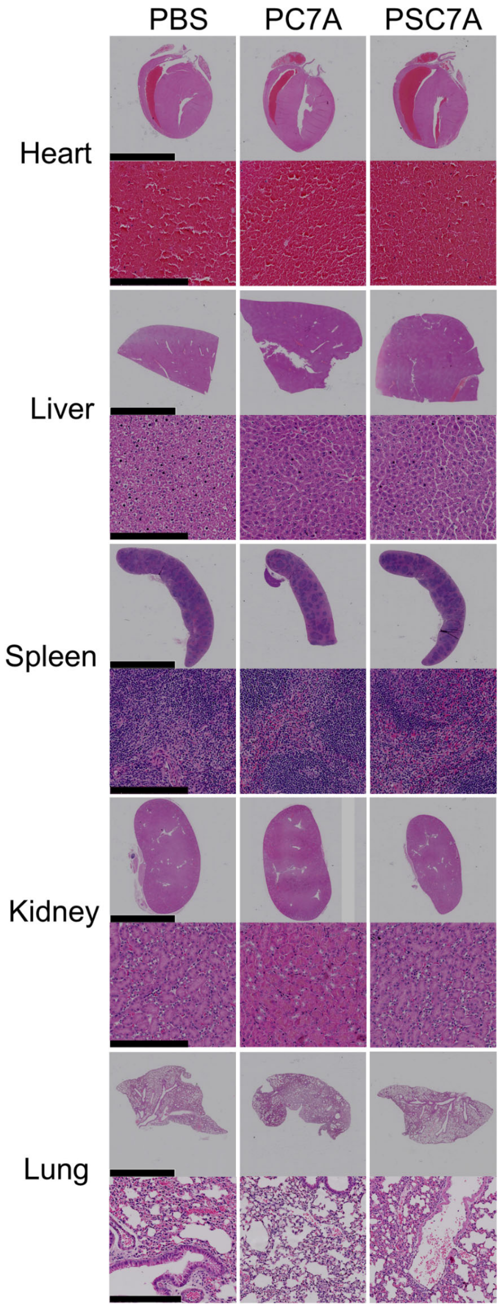

Supplementary Fig. 18 Histologic analysis of pivotal organs for short-term safety evaluation of PSC7A NP. Representative H&E sections of the organs from C57BL/6 mice after subcutaneous injection with PBS, 300 μg PSC7A NP or 300 μg PC7A NP on the right flank. Mice were executed and organs were collected 24 h after administration. Heart, liver, spleen, kidney and lung histology were unremarkable after treatment by either polymer compared to PBS. For each organ group, scale bar: 5 mm (top) and 250 μm (bottom). The experiment was repeated twice with similar results. In each experiment, three biologically independent mice were chosen randomly from each group. A set of organs were harvested from each mouse and five adjacent sections of each set of samples were taken. One set of representative images from each group was shown.

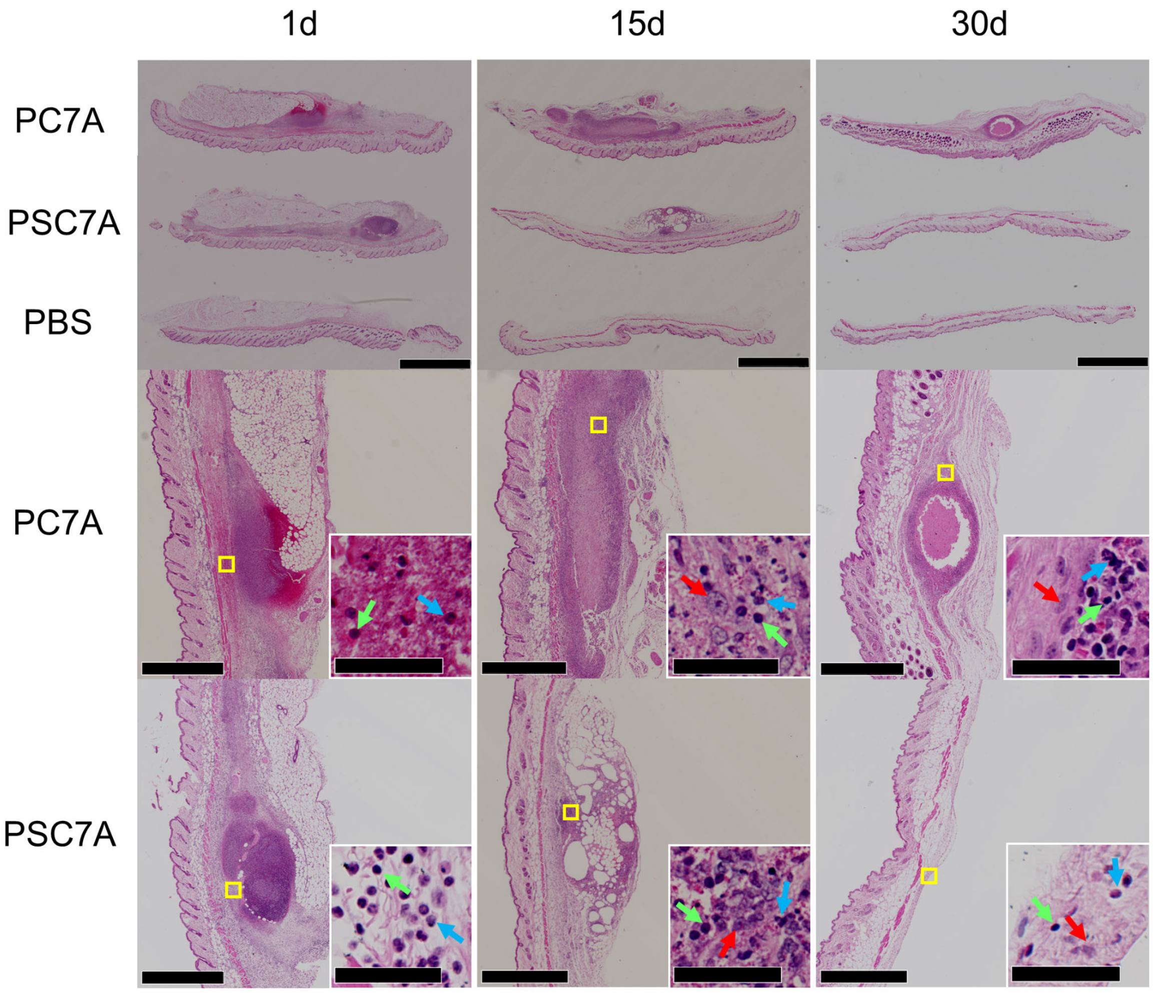

Supplementary Fig. 19 Histologic analysis of the skin tissues (injection sites) for long-term safety evaluation of PSC7A NP. Representative H&E sections of the skin tissues (injection sites) from C57BL/6 mice after subcutaneously injection with PBS, 300 μg PSC7A NP or 300 μg PC7A NP on the right flank. Mice were executed and the skin tissues were collected on day 1, 15 and 30 after administration. Inset: magnification of the area marked by yellow square. The red, green and blue arrows represent macrophages, lymphocytes and neutrophils, respectively. Scale bar: 2.5 mm (top), 1 mm (middle and bottom), 50 μm (inset). The whole set of experiment was repeated twice with similar results. In each experiment, two biologically independent mice were chosen randomly from each group at different time point. Skin tissues were harvested from each mouse and three adjacent sections of each sample were taken. One representative image from each group was shown.
