# Supplementary material for: Polycarbonate-based ultra-pH sensitive nanoparticles improve therapeutic window
Source: Nat Commun. 2020 Nov 17;11:5828. doi: 10.1038/s41467-020-19651-7 (PMC7673035; doi:10.1038/s41467-020-19651-7)
Supplement: Supplementary file 2 — Reporting Summary [file 41467_2020_19651_MOESM2_ESM.pdf]

nature research

Corresponding author(s): Jinming Gao

Last updated by author(s): Oct 8, 2020

Reporting Summary

Nature Research wishes to improve the reproducibility of the work that we publish. This form provides structure for consistency and transparency in reporting. For further information on Nature Research policies, seeAuthors & Referees and theEditorial Policy Checklist .

Statistics

For all statistical analyses, confirm that the following items are present in the figure legend, table legend, main text, or Methods section.

n/a	Confirmed	
☐	☒	The exact sample size (*n*) for each experimental group/condition, given as a discrete number and unit of measurement
☐	☒	A statement on whether measurements were taken from distinct samples or whether the same sample was measured repeatedly
☐	☒	The statistical test(s) used AND whether they are one- or two-sided<br>*Only common tests should be described solely by name; describe more complex techniques in the Methods section.*
☒	☐	A description of all covariates tested
☒	☐	A description of any assumptions or corrections, such as tests of normality and adjustment for multiple comparisons
☐	☒	A full description of the statistical parameters including central tendency (e.g. means) or other basic estimates (e.g. regression coefficient) AND variation (e.g. standard deviation) or associated estimates of uncertainty (e.g. confidence intervals)
☐	☒	For null hypothesis testing, the test statistic (e.g. *F*, *t*, *r*) with confidence intervals, effect sizes, degrees of freedom and *P* value noted<br>*Give P values as exact values whenever suitable.*
☒	☐	For Bayesian analysis, information on the choice of priors and Markov chain Monte Carlo settings
☒	☐	For hierarchical and complex designs, identification of the appropriate level for tests and full reporting of outcomes
☒	☐	Estimates of effect sizes (e.g. Cohen's *d*, Pearson's *r*), indicating how they were calculated

Our web collection on statistics for biologists contains articles on many of the points above.

Software and code

Policy information about availability of computer code

Data collection	NMR spectra of the chemicals were collected on Agilent MR400 or MR500 instruments (UT Southwestern). DLS data were collected on Malvern Nano Zetasizer. TEM images were collected by FEI Tecnai G2 Spirit Biotwin Microscopy. The Fluorescent intensities in the STING reporter experiments were measured by CLARIOstar plate reader. ITC experiments were performed using a Malvern ITC200 microcalorimeter. The titration traces were integrated by NITPIC 1.2.7, and the curves were fitted by SEDPHAT 15.2b. The figures were prepared using GUSSI 1.4.2 (http://biophysics.swmed.edu/MBR/software.html). Flow cytometry data were collected on BD LSRFortessa™ Flow Cytometer. Tissue images were collected by Hamamatsu Nanozoomer 2.0HT.
Data analysis	NMR data were analyzed by Mestrelab's Mnova 8.0.1. Statistical analysis was performed using GraphPad Prism 8.0.1 and Origin8 Pro. For ITC experiment, titration traces were integrated by NITPIC 1.2.7, the curves were fitted by SEDPHAT 15.2b, and the figures were prepared using GUSSI 1.4.2 software. Flow cytometry data were analyzed using BD LSRFortessa™ FACSDiva 8.0.1 software. Tissue images were analyzed by NDP.view 2.7.25.

For manuscripts utilizing custom algorithms or software that are central to the research but not yet described in published literature, software must be made available to editors/reviewers. We strongly encourage code deposition in a community repository (e.g. GitHub). See the Nature Research guidelines for submitting code & software for further information.

Data

Policy information about availability of data

All manuscripts must include a data availability statement. This statement should provide the following information, where applicable:

- Accession codes, unique identifiers, or web links for publicly available datasets
- A list of figures that have associated raw data
- A description of any restrictions on data availability

The authors declare that data supporting the findings of this study are available within the paper and its Supplementary Information files. A reporting summary for this article is available as a Supplementary Information file. The datasets generated and analyzed during the current study are available from the corresponding author upon reasonable request.

Field-specific reporting

Please select the one below that is the best fit for your research. If you are not sure, read the appropriate sections before making your selection.

[x] Life sciences   [ ] Behavioural & social sciences   [ ] Ecological, evolutionary & environmental sciences

For a reference copy of the document with all sections, see nature.com/documents/nr-reporting-summary-flat.pdf

Life sciences study design

All studies must disclose on these points even when the disclosure is negative.

Sample size	Sample sizes were chosen to be capable of obtaining statistical significant differences according to standards of the field and previous publications.
Data exclusions	No data were excluded from the analyses.
Replication	Data reported were consistently replicated across multiple experiments with all replicates generating similar results.
Randomization	For in vivo animal experiments, mice were randomly allocated into each group. For in vitro cell-based experiments, all cells under well controlled conditions were analyzed equally, therefore, no randomization was necessary.
Blinding	Due to practical reasons, true blinding of experiments was not possible in mice treatment administration but they were all under appropriate controls. However, for most of the experiments, data collection and analysis were performed in a blinded manner because each sample was only identified with a number which did't show any information about the treatment administrated.

Reporting for specific materials, systems and methods

We require information from authors about some types of materials, experimental systems and methods used in many studies. Here, indicate whether each material, system or method listed is relevant to your study. If you are not sure if a list item applies to your research, read the appropriate section before selecting a response.

Materials & experimental systems

n/a	Involved in the study	
[ ]	[x]	Antibodies
[ ]	[x]	Eukaryotic cell lines
[x]	[ ]	Palaeontology
[ ]	[x]	Animals and other organisms
[x]	[ ]	Human research participants
[x]	[ ]	Clinical data

Methods

n/a	Involved in the study	
[x]	[ ]	ChIP-seq
[ ]	[x]	Flow cytometry
[x]	[ ]	MRI-based neuroimaging

Antibodies

Antibodies used	Anti-CD3-APC (Biolegend, cat. No. 100235, clone 17A2), anti-CD45-PerCP (Biolegend, cat. no. 103129, clone 30-F11), anti-CD4-FITC (Biolegend, cat. no. 100405, clone GK1.5), anti-CD8-AF700 (Biolegend, cat. no. 100729, clone 53-6.7), anti-H-2Db/HPV16 E7 (RAHYNIVTF) MHC Tetramer-PE (Immudex, cat. no. JA2195), anti-CD11c-FITC (Biolegend, cat. no. 117305, clone N418), anti-CD80-PE/Cy7 (Biolegend, cat. no. 104733, clone 16-10A1).
Validation	All antibody reagents used are commercially available and have been verified by the manufacturers on their websites.

Eukaryotic cell lines

Policy information about cell lines

Cell line source(s)	THP1-ISG cells were provided by Dr. Z. J. Chen (UT Southwestern). B16F10 cells were provided by Dr. Y. X. Fu (UT Southwestern). TC-1 cells were provided by Dr. T. C. Wu (John Hopkins University). B16F10 and TC-1 cells are commercially available from ATCC.
Authentication	The cell lines used in this study were verified by ATCC and monitored for contamination from other cell lines.
Mycoplasma contamination	The cell lines used in this study were free of mycoplasma contamination based on the results of e-Myco Mycoplasma PCR Detection Kit (Bulldog Bio) and were regularly maintained with Normocin.
Commonly misidentified lines (See ICLAC register)	No commonly misidentified cell lines were used.

Animals and other organisms

Policy information about studies involving animals; ARRIVE guidelines recommended for reporting animal research

Laboratory animals	Six- to eight-week-old female wildtype (WT) C57BL/6 mice were obtained from the UTSW Mouse Breeding Core Facility. Six- to eight-week-old female STING knockout (STING-KO) C57BL/6 mice were provided by Dr. Y. X. Fu (UT Southwestern). Mice were housed in a barrier facility with a 12h light/dark cycle and maintained on standard chow (2916 Teklad Global). The temperature range for the housing room is 68-79 ºF (average is around 72 ºF) and the humidity range is 30-50% (average is around 50%).
Wild animals	The study did not involve the use of wild animals.
Field-collected samples	The study did not involve samples collected from the field.
Ethics oversight	All animal experiments were approved by the Institution Animal Care and Use Committees of The University of Texas Southwestern Medical Center and were consistent with local, state, and federal guidelines as applicable.

Note that full information on the approval of the study protocol must also be provided in the manuscript.

Flow Cytometry

Plots

Confirm that:

- [x] The axis labels state the marker and fluorochrome used (e.g. CD4-FITC).
- [x] The axis scales are clearly visible. Include numbers along axes only for bottom left plot of group (a 'group' is an analysis of identical markers).
- [x] All plots are contour plots with outliers or pseudocolor plots.
- [x] A numerical value for number of cells or percentage (with statistics) is provided.

Methodology

Sample preparation	The inguinal lymph nodes of the animals were harvested, ground, filtered, washed and dispersed into single-cell suspensions, and stained with the indicated antibodies for flow cytometry analysis. The primary antibodies used for staining are: anti-CD3-APC (Biolegend, cat. No. 100235, clone 17A2), anti-CD45-PerCP (Biolegend, cat. no. 103129, clone 30-F11), anti-CD4-FITC (Biolegend, cat. no. 100405, clone GK1.5), anti-CD8-AF700 (Biolegend, cat. no. 100729, clone 53-6.7), anti-H-2Db/HPV16 E7 (RAHYNIVTF) MHC Tetramer-PE (Immudex, cat. no. JA2195), anti-CD11c-FITC (Biolegend, cat. no. 117305, clone N418), anti-CD80-PE/Cy7 (Bio-legend, cat. no. 104733, clone 16-10A1).
Instrument	BD LSRFortessa™ Flow Cytometer (BD Biosciences)
Software	BD LSRFortessa™ FACSDiva software
Cell population abundance	The different cell populations were clearly distinguishable and the target cell population (CD8-AF700 and E7 tetramer-PE) was gated as shown in Fig. 3.
Gating strategy	Cells were gated by FSC-SSC > Live/Dead-Aqua negative > CD45-PerCP positive > CD8-AF700 and E7 tetramer-PE double positive

- [ ] Tick this box to confirm that a figure exemplifying the gating strategy is provided in the Supplementary Information.
